# The economic impact of stent retriever selection for acute ischemic stroke: a cost analysis of MASTRO I from the healthcare system perspective of the United States, Canada and eight European countries

**DOI:** 10.57264/cer-2024-0216

**Published:** 2025-02-17

**Authors:** Tommy Andersson, Hannes Nordmeyer, Waleed Brinjikji, Emilie Kottenmeier, Mina Kabiri, Shanti Scheffler, Patrick A Brouwer, Mahmood Mirza, Osama O Zaidat

**Affiliations:** 1 Medical Imaging, AZ Groeninge, 8500, Kortrijk, Belgium; 2 Neuroradiology, Karolinska University Hospital & Clinical Neuroscience Karolinska Institutet, 171 77, Stockholm, Sweden; 3 Department of Neuroradiology, Klinikum Solingen, Solingen, Germany; 4 School of Medicine, Department of Health, Witten/Herdecke University, Witten, Germany; 5 Department of Radiology, Mayo Clinic, Rochester, MN 55902, USA; 6 Johnson & Johnson MedTech, Irvine, CA 92618, USA; 7 Johnson & Johnson MedTech, Global Health Economics & Market Access, Raynham, MA 02767, USA; 8 Johnson & Johnson MedTech, 6300 Zug, Switzerland; 9 Mercy St Vincent Medical Center, Toledo, OH 43608, USA

**Keywords:** acute ischemic stroke, cost, economic, EmboTrap, MASTRO I, mechanical thrombectomy, modified Rankin Scale, Solitaire, stent retriever, Trevo

## Abstract

**Aim:**

According to the results of the MASTRO I living systematic review and meta-analysis, use of the EmboTrap Revascularization^®^ Device in the treatment of acute ischemic stroke (AIS) results in higher rates of good functional outcomes (90-day modified Rankin Scale [mRS] 0–2) compared with use of the Trevo^®^ Retriever or the Solitaire™ Revascularization Device. The aim of this analysis was to assess the potential economic impact of achieving improved functional outcomes for three commonly used stent retrievers (SRs) in the treatment of AIS.

**Methods:**

An economic model with short-term and long-term costs, representing a healthcare system perspective was developed using a decision tree to simulate a cohort of 1000 hypothetical patients treated for AIS with mechanical thrombectomy (MT) using EmboTrap, Trevo or Solitaire SRs. Based on the proportion of patients who achieved a 90-day mRS score of 0–2 or 3–5 for each device reported in MASTRO I (excluding patients not surviving after 90 days), this model estimated per-patient costs and the associated incremental cost savings. Results are reported from the healthcare system perspective in the US, Canada, the UK, Sweden, Germany, France, Italy, Spain, Belgium and The Netherlands.

**Results:**

Across all ten countries, the use of EmboTrap during MT was associated with the lowest short-term (ranging from €8412 in Italy to $66,525 in the US), long-term (ranging from €5249 in Italy to $25,757 in the US) and total (ranging from €13,661 in Italy to $92,282 in the US) per-patient costs. The total per-patient cost was higher with Trevo (ranging from €14,601 in Italy to $97,487 in the US) and Solitaire (ranging from €14,840 in Italy to $98,814 in the US). Cost savings were highest when comparing EmboTrap versus Solitaire, followed by EmboTrap versus Trevo, with Trevo versus Solitaire having the smallest cost savings. Results of sensitivity and scenario analyses supported the robustness of the base-case results.

**Conclusion:**

Across the ten countries, treating patients with AIS with EmboTrap resulted in lower short-term, long-term and total costs to the payer. With rising healthcare costs and limited hospital budgets, these results suggest EmboTrap proves to be an evidence-based economical choice of SR for hospitals and healthcare systems.

## Plain language summary

**What is this article about?:**

Mechanical thrombectomy (MT) is the current standard of care for the treatment of acute ischemic stroke and can be performed using stent retrievers (SRs), aspiration catheters or a combination of both. MASTRO I, a recently published systematic review and meta-analysis, compared outcomes following MT for three commonly used SRs (EmboTrap Revascularization^®^ Device, Trevo^®^ Retriever and Solitaire™ Revascularization Device) and found that the SR used during MT can impact functional outcomes. Data from MASTRO I reported that compared with Trevo and Solitaire, treatment with EmboTrap was associated with improved functional outcomes. As better functional outcomes may result in lower healthcare resource utilization, SR choice during MT may have downstream economic implications. Therefore, the aim of the current analysis was to compare the total (short- and long-term) economic outcomes with the use of EmboTrap, Trevo and Solitaire in the US, Canada, the UK, Sweden, Germany, France, Italy, Spain, Belgium and The Netherlands.

**What were the results?:**

EmboTrap use was associated with the lowest short-term, long-term and total costs in all ten countries evaluated, followed by Trevo and Solitaire.

**What do the results mean?:**

These results may assist physicians and hospital stakeholders in supporting evidence-based decision making when selecting an optimal choice of SR for MT. The results from this analysis suggest that, beyond device cost, optimizing patient functional outcomes through device selection can impact downstream economic consequences across all ten countries studied.

## Graphical abstract

**Figure:**
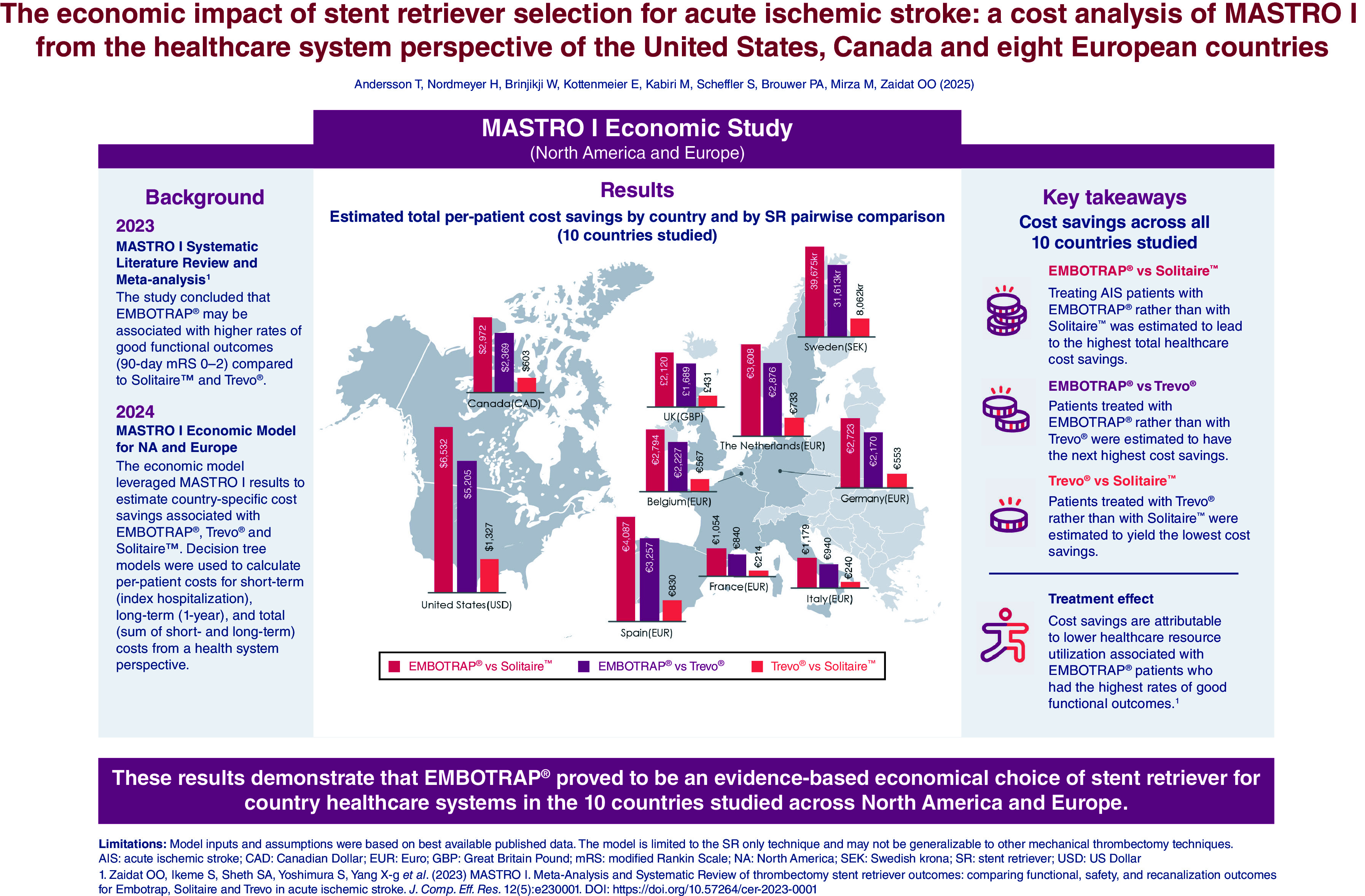

Stroke is a leading cause of death and disability worldwide [1,2,3], and survivors often experience long-term deficits, with approximately 90% experiencing disability [4]. As a result, patients, their caregivers [5,6] and healthcare systems [1] are substantially impacted by the disease.

Total direct medical costs related to stroke are projected to more than double by 2035 due to the global aging population particularly across high-income countries [1], which will significantly increase the already substantial economic burden of stroke. Care for patients who have experienced a stroke was estimated to cost the US healthcare system $56.2 billion USD in direct and indirect costs between 2019 and 2020, with the direct cost of stroke estimated to be $34.5 billion USD [7]. In Canada, the total stroke-related healthcare costs in the first year following stroke was estimated to be $2 billion Canadian dollars (CAD) in 2021 [8]. Across Europe, stroke-related healthcare costs were estimated to be €27 billion in 2017 [9]. Therefore, opportunities to reduce stroke-related costs should be prioritized for consideration.

Approximately 63–87% of all strokes are ischemic [1,3], with 31–46% caused by a large vessel occlusion (LVO) [10,11,12]. Mechanical thrombectomy (MT) is the current standard of care for eligible patients with acute ischemic stroke (AIS) due to LVO [13,14,15]. Compared with standard of care of intravenous thrombolysis alone, MT results in better clinical outcomes [13,16], including good functional outcomes defined by modified Rankin Scale (mRS) score of 0–2 at 90-days post-stroke [13,16], and is more cost-effective [17,18]. During MT, different techniques and devices, such as aspiration catheters, stent retrievers (SRs) or a combination of both, can be used to restore blood flow. The use of SRs is well established and recommended in clinical guidelines [13,14,15], however individual SRs possess unique design characteristics and mechanisms known to influence recanalization and functional outcomes during and following MT [19,20,21,22].

The results of a recent systematic literature review and meta-analysis, MASTRO I, suggest that SR choice may impact functional outcomes following MT with a SR used as the first-line technique [20]. The use of the EmboTrap Revascularization^®^ Device during MT resulted in significantly higher rates of 90-day mRS 0–2 (57.4%) compared with the use of the Solitaire™ Revascularization Device (45.3%, p < 0.001) or the Trevo^®^ Retriever (50.0%, p = 0.013) [20]. As post-stroke mRS score has been shown to directly correlate with medical costs, patients with 90-day mRS 3–5 (i.e., functionally dependent) are likely to have higher healthcare-related costs than patients with 90-day mRS 0–2 (i.e., functionally independent) [23,24].

Currently, no published research has estimated the economic impact of SR selection following MT for AIS. The aim of this analysis was to assess the economic impact of achieving improved functional outcomes with the use of three commonly used SRs for ten countries from the healthcare system perspective.

## Methods

### Study design

The cost model was designed to compare the cost of using EmboTrap, Trevo and Solitaire, based on their differential proportion of patients in mRS 0–2 versus mRS 3–5 at 90 days post MT. The model assessed one-year costs from the healthcare systems perspective for ten countries: the US, Canada, the UK, Sweden, Germany, France, Italy, Spain, Belgium and The Netherlands. Costs included index hospitalization costs as well as post-discharge healthcare costs up to 1-year post-stroke. The details of cost outcomes are presented in the following sections.

### Model structure

**Figure 1.**
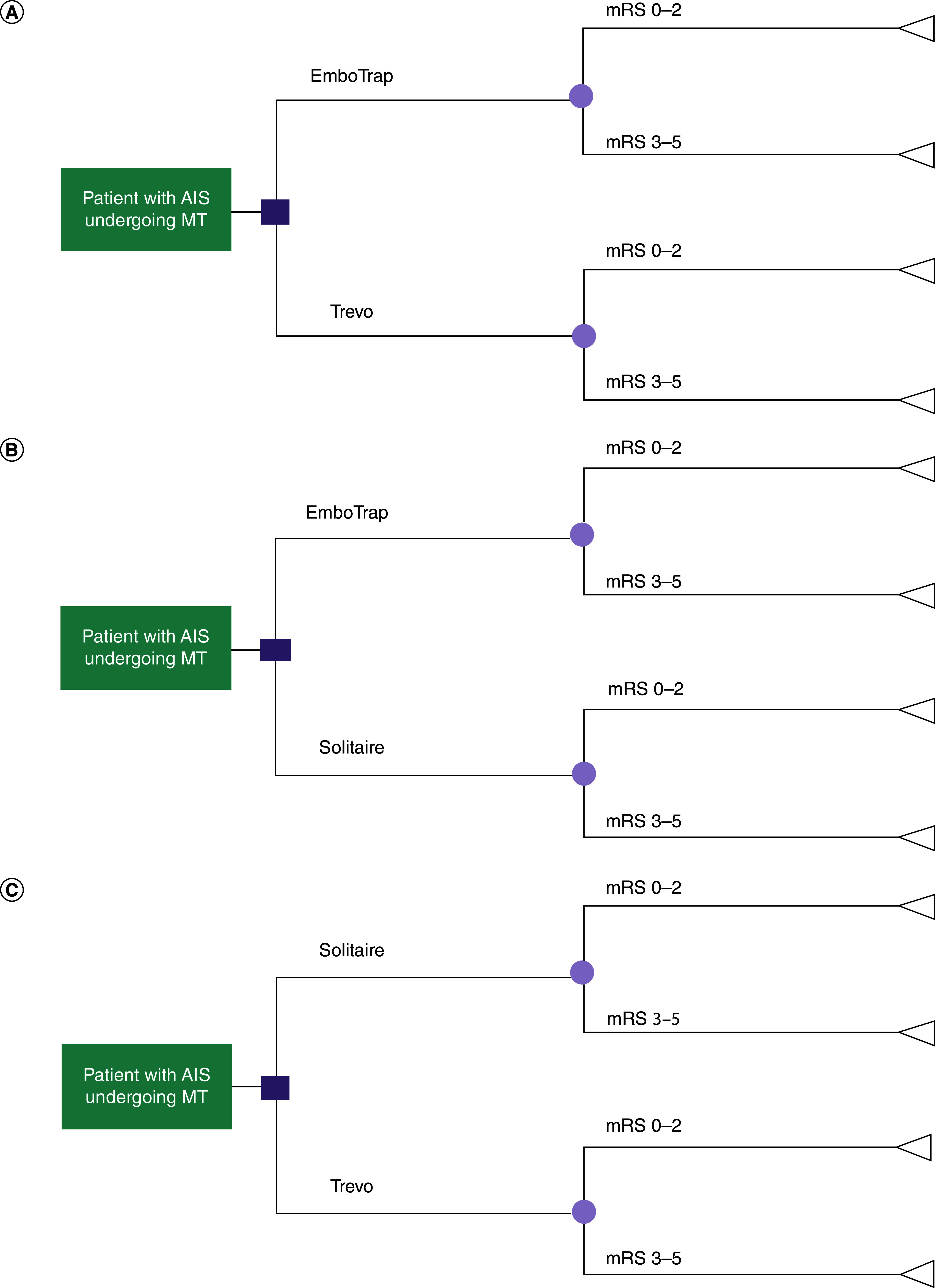
Decision trees for stent retriever selection in treating acute ischemic stroke. The decision tree depicts possible branches of treatments for the treatment of AIS for patients who were treated with **(A)** EmboTrap versus Trevo, **(B)** EmboTrap versus Solitaire, **(C)** Solitaire versus Trevo and were alive 90-days post-treatment. The square represents the node between receiving treatment with EmboTrap and either Solitaire or Trevo. The circle represents the node between the two possible mRS scores the patient can achieve: mRS 0–2 or 3–5. The triangle represents the economic outcomes associated with achieving the mRS score. AIS: Acute ischemic stroke; mRS: modified Rankin Scale; MT: Mechanical thrombectomy; SR: Stent retriever.

Three decision trees were developed per country using R (version 3.6.1) for three pairwise comparisons: EmboTrap versus Trevo, EmboTrap versus Solitaire, and Trevo versus Solitaire. Each decision tree simulated a cohort of 1000 adult patients with AIS due to emergent large vessel occlusion treated with MT using either EmboTrap, Trevo or Solitaire as the first-line SR and included two health states: good functional outcomes (90-day mRS 0–2) or poor functional outcomes (90-day mRS 3–5) (Figure 1). Patient cohort represented the pooled patient characteristics, such as age, sex and recanalization outcomes, as reported in MASTRO I [20].

### Clinical efficacy & cost inputs

Clinical efficacy inputs included the proportion of patients who achieved good functional outcomes (90-day mRS 0–2) versus poor functional outcomes (90-day mRS 3–5) by device obtained from MASTRO I [20] (Table 1). We assumed all other clinical efficacy components related to SR devices were similar across patient cohorts.

**Table 1.** Model inputs and probabilistic sensitivity analyses distributions.

Input parameter		Value	Probability distribution and parameters	Source (year)	Ref.
Distribution of patients by mRS category^‡^					
EmboTrap					
mRS 0–2		64.64%	Multinomial (0.574, 0.314, 0.112)	Zaidat *et al.* (2023)	[20]
mRS 3–5		35.36%			
Trevo					
mRS 0–2		58.48%	Multinomial (0.500, 0.355, 0.145)	Zaidat *et al.* (2023)	[20]
mRS 3–5		41.52%			
Solitaire					
mRS 0–2		56.91%	Multinomial (0.453, 0.343, 0.204)	Zaidat *et al.* (2023)	[20]
mRS 3–5		43.09%			
**Short-term index hospitalization inputs**					
Mean index hospitalization length of stay (days)					
mRS 0–2		8.9	Gamma^†^ (α = 168.66, β = 19.052)	Zaidat *et al.* (2023) and Dewilde *et al.* (2017)	[20] and [24]
mRS 3–5		18.4	Gamma (α = 168.66, β = 9.240)		
Index hospitalization cost per day (2023 values) by country					
US (USD)		$5433	Gamma^†^ (α = 168.66, β = 0.0312)	Simpson *et al.* (2017)	[25]
Canada (CAD)		$1315	Gamma^†^ (α = 168.66, β = 0.1290)	Mittmann *et al.* (2012)	[26]
UK (GBP)		£766	Gamma^†^ (α = 168.65, β = 0.2214)	Luengo-Fernandez *et al.* (2020)	[9]
Sweden (SEK)		11,578kr	Gamma^†^ (α = 168.66, β = 0.1656)	Luengo-Fernandez *et al.* (2020)	[9]
Germany (EUR)		€950	Gamma^†^ (α = 168.69, β = 0.1786)	Luengo-Fernandez *et al.* (2020)	[9]
France (EUR)		€690	Gamma^†^ (α = 168.65, β = 0.2458)	Luengo-Fernandez *et al.* (2020)	[9]
Italy (EUR)		€687	Gamma^†^ (α = 168.65, β = 0.2469)	Luengo-Fernandez *et al.* (2020)	[9]
Spain (EUR)		€758	Gamma^†^ (α = 168.66, β = 0.2237)	Luengo-Fernandez *et al.* (2020)	[9]
Belgium (EUR)		€1028	Gamma^†^ (α = 168.65, β = 0.1650)	Luengo-Fernandez *et al.* (2020)	[9]
The Netherlands (EUR)		€1674	Gamma^†^ (α = 168.66, β = 0.1013)	Luengo-Fernandez *et al.* (2020)	[9]
**Long-term 1-year post-index hospitalization inputs**					
1-year post-stroke care costs (2023 values) by country					
US (USD)	mRS 0–2	$14,038	Gamma^†^ (α = 168.67, β = 0.0120)	Shireman *et al.* (2017)	[18]
	mRS 3–5	$47,180	Gamma^†^ (α = 168.68, β = 0.0036)		
Canada (CAD)	mRS 0–2	$19,506	Gamma^†^ (α = 168.68, β = 0.0087)	Mittmann *et al.* (2012)	[26]
	mRS 3–5	$45,525	Gamma^†^ (α = 168.68, β = 0.0037)		
UK (GBP)	mRS 0–2	£4452	Gamma^†^ (α = 168.66, β = 0.0381)	Lobotesis *et al.* (2016)	[27]
	mRS 3–5	£24,632	Gamma^†^ (α = 168.68, β = 0.0069)		
Sweden (SEK)	mRS 0–2	62,208kr	Gamma^†^ (α = 168.66, β = 0.0308)	Lekander *et al.* (2017)	[32]
	mRS 3–5	466,020kr	Gamma^†^ (α = 168.69, β = 0.0041)		
Germany (EUR)	mRS 0–2	€2049	Gamma^†^ (α = 168.66, β = 0.0828)	Oliveira Gonçalves *et al.* (2023)	[28]
	mRS 3–5	€28,297	Gamma^†^ (α = 168.69, β = 0.0060)		
France (EUR)	mRS 0–2	€7901	Gamma^†^ (α = 168.66, β = 0.0215)	Barral *et al.* (2020)	[29]
	mRS 3–5	€15,013	Gamma^†^ (α = 168.67, β = 0.0113)		
Italy (EUR)	mRS 0–2	€2150	Gamma^†^ (α = 168.65, β = 0.0789)	Fattore *et al.* (2012)	[30]
	mRS 3–5	€10,913	Gamma^†^ (α = 168.67, β = 0.0116)		
Spain (EUR)	mRS 0–2	€3177	Gamma^†^ (α = 168.65, β = 0.0534)	de Andrés-Nogales *et al.* (2017)	[31]
	mRS 3–5	€48,887	Gamma^†^ (α = 168.68, β = 0.0347)		
Belgium (EUR)	mRS 0–2	€4510	Gamma^†^ (α = 168.65, β = 0.0458)	Dewilde *et al.* (2017)	[24]
	mRS 3–5	€30,937	Gamma^†^ (α = 168.68, β = 0.0067)		
The Netherlands (EUR)	mRS 0–2	€7067	Gamma^†^ (α = 168.66, β = 0.0240)	Pinckaers *et al.* (2024)	[33]
	mRS 3–5	€37,923	Gamma^†^ (α = 168.69, β = 0.0045)		

† α and β present shape and rate parameters for inputs following a Gamma distribution and are rounded to two and four decimal points, respectively.

‡ Data reweighted to exclude mRS 6.

CAD: Canadian dollar; EUR: Euro; GBP: Great Britain Pound; mRS: 90-day modified Rankin Scale score; NA: Not applicable; NR: not reported; SEK: Swedish krona; USD: United States dollar.

This analysis included costs obtained from published literature. Cost inputs did not differ by choice of SR device, leaving clinical efficacy inputs as the driver of model outcomes.

Short-term cost inputs were calculated for mRS 0–2 and 3–5 health states by multiplying the weighted average length of stay (LOS) by the per-patient cost for hospital day following stroke [9,25,26] for each state. We calculated the weighted average LOS for mRS 0–2 and 3–5 health states by aggregating the distribution of patient volume across mRS 0–5 categories and their respective average LOS reported in the literature [20,24]. Device cost was not included in the model.

Weighted average long-term cost inputs for mRS 0–2 and 3–5 states were calculated using the per-patient long-term costs by 90-day mRS score (0–5) [18,24,26–33], aggregated using the distribution of patients in 90-day mRS score (0–5) [20]. Per-patient costs by 90-day mRS score were obtained from published literature (Table 1).

**Figure 2.**
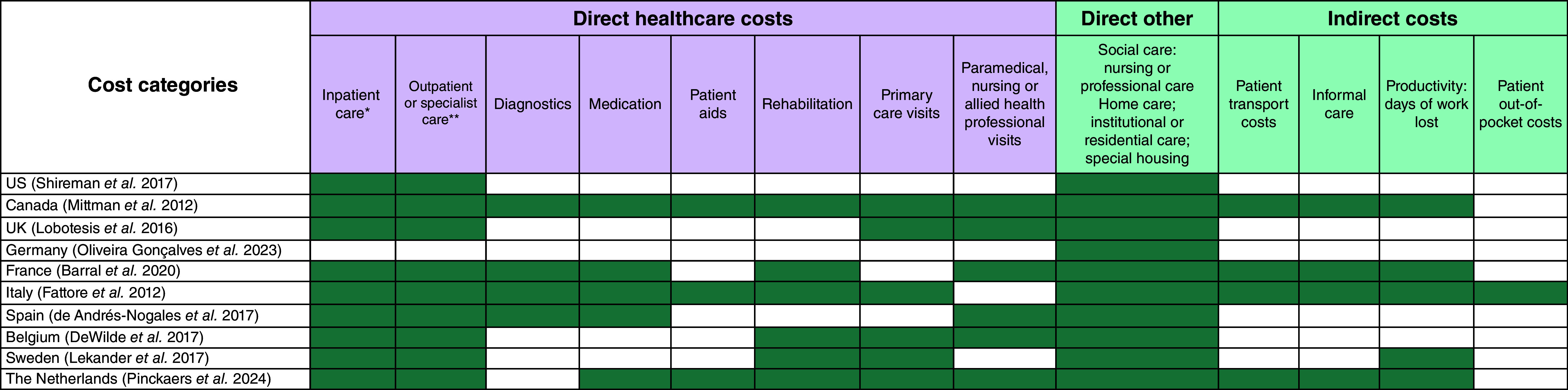
Comparison of country-specific long-term costs included in the model. The figure depicts the cost categories that are included in the country-specific long-term costs included in the model. Green cells indicate that the cost is included in the long-term cost input used in the model. *Includes visits to emergency room. **Also includes day cases.

Costs captured in the long-term cost inputs included direct long-term care costs and social care (direct other) costs, the latter of which reflected nursing or professional care, home care, institutional or residential care, and/or special housing costs. Where possible, indirect costs were removed from the long-term cost inputs. Figure 2 outlines the long-term cost categories reported for each country by source. Due to limitations in long-term data reported for Canada, Germany, France and The Netherlands, additional assumptions were required to impute direct long-term care costs (Supplementary Methods).

Costs for the US, Canada, European countries and the UK were included in the model in local currency (i.e., USD, CAD, Euros and Great Britain Pounds [GBP], respectively). Belgian costs were sourced from a publication that reported costs in USD [24], thus costs were converted to Euros [34]. Costs for Sweden were reported in Euros from the source publication [32]. Unless otherwise noted, costs for all countries were inflated to 2023 values using the health-specific Consumer Price Indices from the International Monetary Fund [35]. The cost inputs in the model were assumed to be the same regardless of the SR device used (Table 1).

### Model outcomes

The cost model estimated per-patient cost savings (incremental costs) for each country based on the pairwise device comparisons of total costs, comprised of short- and long-term costs, described below. In addition to per-patient incremental cost outcomes, the percent differences in total cost for each pairwise comparison were calculated to provide a normalized assessment of cost savings. Cost outcomes were reported in the same currency they were input into the model, with the exception of Sweden where results are reported in local currency (2023 Swedish Krona [SEK]).

**Figure 3.**
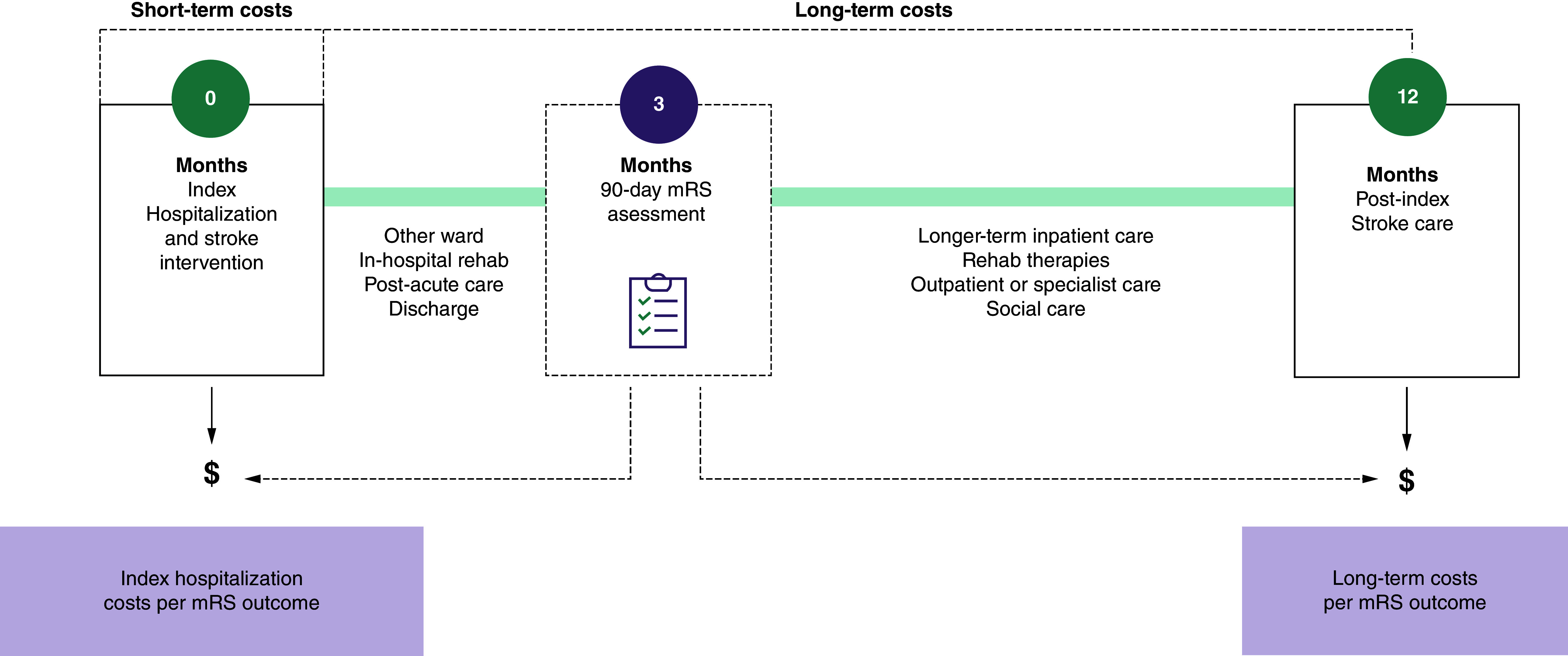
Overall time horizon of the short- and long-term analyses. mRS: 90-day modified Rankin Scale score.

Short-term costs included the costs accrued during index hospitalization after the MT procedure. Per-patient short-term costs were calculated using the average per-patient cost of mRS 0–2 and 3–5 states for hospitalization LOS multiplied by their respective proportion of patients in each state for each device. Long-term costs included the healthcare costs accrued from the time of patient discharge to one-year post-stroke. Per-patient long-term costs were calculated using the average per-patient cost of mRS 0–2 and 3–5 states for 1 year post MT weighted by the device-specific proportion of patients in each state. Figure 3 depicts the overall time horizon for the cost model.

As cost estimates for death (mRS 6) were not consistently reported in the source publications, this analysis focused on patients who survived to 90 days. Device-specific patient volume for mRS 0–2 and 3-5 were proportionally adjusted to exclude mRS 6 and preserve differences in treatment effect (Table 1).

### Sensitivity & scenario analyses

Probabilistic sensitivity analyses were conducted based on 10,000 iterations of parametric Monte Carlo simulations. Input parameters varied in each iteration of the probabilistic sensitivity analyses according to the probability distributions reported in Table 1. 95% credible intervals (CrIs) were estimated for all model outcomes. Due to the hypothetical patient population used in this model, Crls are used to represent the range between which 95% of probabilistic sensitivity analysis results fall.

Deterministic one-way sensitivity analyses were performed for each country by varying individual input parameters by ± 15%. The proportional distribution of patients across mRS categories was conserved to preserve logic and ensure patient percentages always summed to 100%.

A probabilistic scenario analysis was conducted based on the statistical outlier analysis performed in MASTRO I [20], which excluded the studies that reported values that differed significantly from the overall effect in the meta-analysis. In this scenario analysis, the proportion of patients that achieved mRS 0–2 at 90 days was reduced for EmboTrap (from 57.4 to 54.9%), increased for Solitaire (from 45.3 to 46.2%) and did not change for Trevo (50.0%) [20].

## Results

### Deterministic base-case analyses

Across all ten countries, patients treated with EmboTrap were estimated to have the lowest short-term costs, long-term costs and total costs. Costs were higher in the cohort treated with Trevo, and highest in the cohort treated with Solitaire (Table 2). The estimated cost savings over the one-year period were greatest for EmboTrap versus Solitaire, followed by EmboTrap versus Trevo, with the smallest cost savings reported between Trevo versus Solitaire.

**Table 2.** Deterministic base-case results for EmboTrap, Trevo and Solitaire.

Country and device	Short-term cost	Long-term cost	Total cost	Per-patient incremental cost compared with EmboTrap	Per-patient incremental cost compared with Trevo
**US (USD)**					
EmboTrap	$66,525	$25,757	$92,282	–	–
Trevo	$69,689	$27,799	$97,487	$5205	–
Solitaire	$70,495	$28,319	$98,814	$6532	$1327
**Canada (CAD)**					
EmboTrap	$16,102	$28,707	$44,808	–	–
Trevo	$16,868	$30,309	$47,177	$2369	–
Solitaire	$17,063	$30,718	$47,780	$2972	$603
**UK (GBP)**					
EmboTrap	£9380	£11,588	£20,967		–
Trevo	£9826	£12,830	£22,656	£1689	–
Solitaire	£9940	£13,147	£23,087	£2120	£431
**Sweden (SEK)**					
EmboTrap	141,761kr	204,998kr	346,770kr	–	–
Trevo	148,511kr	229,884kr	378,383kr	31,613kr	–
Solitaire	138,923kr	236,215kr	386,445kr	39,675kr	8062kr
**Germany (EUR)**					
EmboTrap	€11,632	€11,330	€22,963	–	–
Trevo	€12,186	€12,947	€25,133	€2170	–
Solitaire	€12,327	€13,359	€25,686	€2723	€553
**France (EUR)**					
EmboTrap	€8448	€10,416	€18,865	–	–
Trevo	€8851	€10,853	€19,705	€840	–
Solitaire	€8953	€10,965	€19,919	€1054	€214
**Italy (EUR)**					
EmboTrap	€8412	€5249	€13,661	–	–
Trevo	€8812	€5788	€14,601	€940	–
Solitaire	€8914	€5927	€14,840	€1179	€240
**Spain (EUR)**					
EmboTrap	€9282	€19,340	€28,621	–	–
Trevo	€9723	€22,156	€31,879	€3257	–
Solitaire	€9835	€22,874	€32,709	€4087	€830
**Belgium (EUR)**					
EmboTrap	€12,587	€13,854	€26,442	–	–
Trevo	€13,187	€15,482	€28,668	€2227	–
Solitaire	€13,339	€15,897	€29,236	€2794	€567
**The Netherlands (EUR)**					
EmboTrap	€20,497	€17,979	€38,476	–	–
Trevo	€21,473	€19,879	€41,351	€2876	–
Solitaire	€21,721	€20,363	€42,084	€3608	€733

Results for Germany represent both 2023 and 2024 values due to the use of 2024 physician fees to calculate long-term costs.

CAD: Canadian dollar; EUR: Euro; GBP: Great Britain Pound; SEK: Swedish krona; USD: United States dollar.

As outlined in Table 2, compared with Trevo, EmboTrap was estimated to have per-patient incremental total cost savings of USD $5205 in the US, CAD $2369 in Canada, £1689 in the UK, SEK 31,613kr in Sweden, €2170 in Germany, €840 in France, €940 in Italy, €3257 in Spain, €2227 in Belgium and €2876 in The Netherlands.

Compared with Solitaire, EmboTrap was estimated to have per-patient incremental total cost savings of USD $6532 in the US, CAD $2972 in Canada, £2120 in the UK, SEK 39,675kr in Sweden, €2723 in Germany, €1054 in France, €1179 in Italy, €4087 in Spain, €2794 in Belgium and €3608 in The Netherlands (Table 2).

Relative to Solitaire, Trevo was estimated to have per-patient incremental total cost savings of USD $1327 in the US, CAD $603 in Canada, £431 in the UK, SEK 8062kr in Sweden, €553 in Germany, €214 in France, €240 in Italy, €830 in Spain, €567 in Belgium and €733 in The Netherlands (Table 2).

**Figure 4.**
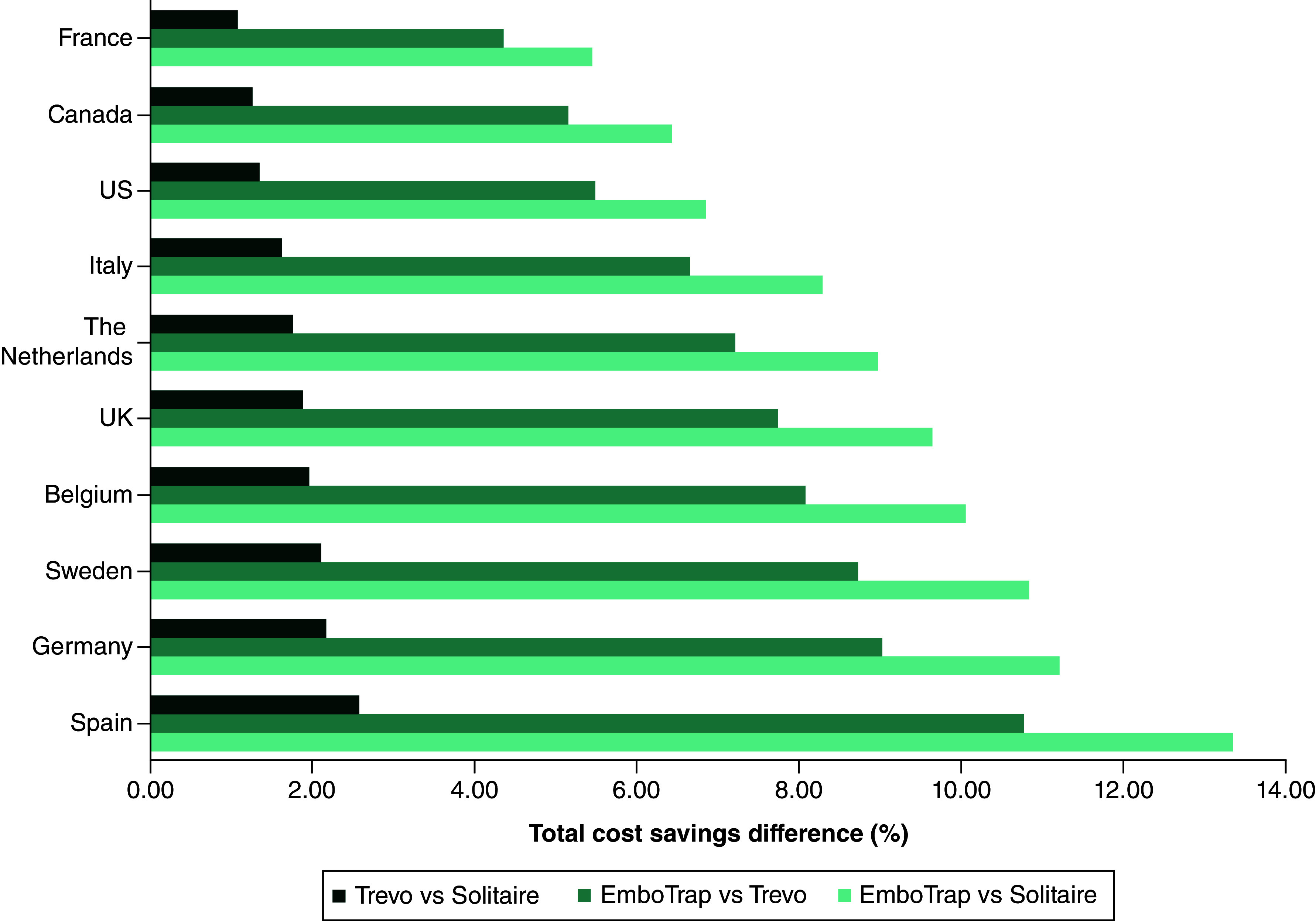
Per-patient pairwise comparison of total cost savings.

The normalized assessment of total cost, demonstrated by the percent difference in total costs, indicates that the greatest magnitude of cost savings for all pairwise comparisons was estimated for Spain and Germany, with France having the lowest cost savings (Figure 4).

### Probabilistic sensitivity analyses

The probabilistic sensitivity analyses aligned with the deterministic base-case results, with EmboTrap yielding the lowest short-term, long-term and total costs. Trevo, followed by Solitaire, had higher costs. Like the base-case, cost savings were the highest for EmboTrap versus Solitaire, followed by EmboTrap versus Trevo, with Trevo versus Solitaire yielding the lowest cost savings (Table 3).

**Table 3.** Economic outcomes of probabilistic sensitivity analyses for EmboTrap, Trevo and Solitaire.

Country and device	Short-term cost Mean (95% CrI)	Long-term cost Mean (95% CrI)	Total cost Mean (95% CrI)	Per-patient incremental cost compared with EmboTrap Mean (95% CrI)	Iterations with cost savings using EmboTrap	Per-patient incremental cost compared with Trevo Mean (95% CrI)	Iterations with cost savings using Trevo
**US (USD)**							
EmboTrap	$65,797 (65,674; 65,920)	$25,637 (25,607; 25,668)	$91,434 (91,306; 91,562)	–	–	–	–
Trevo	$68,925 (68,795; 69,054)	$27,673 (27,639; 27,706)	$96,597 (96,463; 96,732)	$5163 (4580; 5747)	99.62%	–	–
Solitaire	$69,729 (69,598; 69,861)	$28,199 (28,164; 28,233)	$97,928 (97,790; 98,066)	$6494 (5900; 7088)	99.92%	$1331 (722; 1939)	74.92%
**Canada (CAD)**							
EmboTrap	$15,925 (15,896; 15,955)	$28,576 (28,545; 28,608)	$44,502 (44,458; 44,546)	–	–	–	–
Trevo	$16,682 (16,651; 16,714)	$30,174 (30,140; 30,208)	$46,856 (46,810; 46,903)	$2354 (2153; 2557)	99.62%	–	–
Solitaire	$16,877 (16,845; 16,909)	$30,587 (30,552; 30,622)	$47,464 (47,416; 47,513)	$2962 (2756; 3169)	99.92%	$608 (395; 820)	74.92%
**UK (GBP)**							
EmboTrap	£9277 (9259; 9294)	£11,532 (11,517; 11,547)	£20,809 (20,785; 20,832)	–	–	–	–
Trevo	£9718 (9699; 9736)	£12,772 (12,755; 12,789)	£22,489 (22,464; 22,515)	£1680 (1571; 1790)	99.62%	–	–
Solitaire	£9831 (9813; 9850)	£13,092 (13,074; 13,110)	£22,923 (22,896; 22,949)	£2114 (2002; 2226)	99.92%	£434 (317; 550)	74.92%
**Sweden (SEK)**							
EmboTrap	140,212kr (139,952; 140,472)	204,003kr (203,721; 204,286)	344,215kr (343,819; 344,622)	–	–	–	–
Trevo	146,883kr (146,600; 147,154)	228,810kr (228,482; 229,126)	375,681kr (375,251; 376,122)	31,466kr (29,623; 33,309)	99.62%	–	–
Solitaire	148,590kr (148,319; 148,873)	235,209kr (234,870; 235,548)	383,810kr (383,358; 384,263)	39,584kr (37,685; 41,484)	99.92%	8118kr (6139; 10,097)	74.92%
**Germany (EUR)**							
EmboTrap	€11,505 (11,484; 11,527)	€11,274 (11,257; 11,292)	€22,779 (22,751; 22,808)	–	–	–	–
Trevo	€12,052 (12,029; 12,075)	€12,886 (12,867; 12,906)	€24,938 (24,908; 24,969)	€2159 (2028; 2290)	99.62%	–	–
Solitaire	€12,193 (12,170; 12,216)	€13,303 (13,282; 13,323)	€25,495 (25,463; 25,527)	€2716 (2581; 2851)	99.92%	€557 (417; 697)	74.92%
**France (EUR)**							
EmboTrap	€8356 (8341; 8372)	€10,369 (10,358; 10,381)	€18,726 (18,706; 18,745)	–	–	–	–
Trevo	€8754 (8737; 8770)	€10,806 (10,794; 10,818)	€19,560 (19,539; 19,580)	€834 (745; 923)	99.62%	–	–
Solitaire	€8856 (8839; 8872)	€10,919 (10,907; 10,931)	€19,775 (19,754; 19,796)	€1049 (959; 1139)	99.92%	€215 (123; 307)	74.92%
**Italy (EUR)**							
EmboTrap	€8320 (8304; 8336)	€5224 (5217; 5231)	€13,544 (13,527; 13,561)	–	–	–	–
Trevo	€8715 (8699; 8732)	€5762 (5754; 5770)	€14,477 (14,459; 14,496)	€934 (855; 1013)	99.62%	–	–
Solitaire	€8817 (8801; 8834)	€5901 (5893; 5909)	€14,718 (14,699; 14,737)	€1174 (1094; 1255)	99.92%	€241 (158; 324)	74.92%
**Spain (EUR)**							
EmboTrap	€9167 (9150; 9184)	€19,226 (19,196; 19,256)	€28,393 (28,358; 28,428)	–	–	–	–
Trevo	€9603 (9585; 9621)	€22,025 (21,991; 22,060)	€31,628 (31,589; 31,668)	€3235 (3068; 3402)	99.59%	–	–
Solitaire	€9714 (9696; 9733)	€22,742 (22,707; 22,778)	€32,456 (32,416; 32,497)	€4063 (3893; 4234)	99.92%	€828 (648; 1009)	73.98%
**Belgium (EUR)**							
EmboTrap	€12,450 (12,426; 12,473)	€13,788 (13,769; 13,806)	€26,237 (26,206; 26,268)	–	–	–	–
Trevo	€13,042 (13,017; 13,066)	€15,411 (15,389; 15,432)	€28,452 (28,419; 28,485)	€2215 (2073; 2357)	99.62%	–	–
Solitaire	€13,194 (13,169; 13,219)	€15,830 (15,807; 15,852)	€29,024 (28,989; 29,058)	€2786 (2640; 2932)	99.92%	€571 (420; 723)	74.92%
**The Netherlands (EUR)**							
EmboTrap	€20,267 (20,230; 20,305)	€17,879 (17,856; 17,902)	€38,146 (38,102; 38,191)	–	–	–	–
Trevo	€21,235 (21,196; 21,274)	€19,775 (19,749; 19,801)	€41,010 (40,962; 41,058)	€2864 (2658; 3070)	99.64%	–	–
Solitaire	€21,478 (21,438; 21,517)	€20,251 (20,224; 20,278)	€41,729 (41,680; 41,778)	€3583 (3373; 3793)	99.90%	€719 (502; 936)	73.67%

Probabilistic analyses were based on parametric Monte Carlo simulations with 10,000 iterations.

Results for Germany represent both 2023 and 2024 values due to the use of 2024 physician fees to calculate long-term costs.

CAD: Canadian dollar; CrI: Credible interval; EUR: Euro; GBP: Great Britain Pound; SEK: Swedish krona; USD: United States dollar.

As outlined in Table 3, compared with Trevo, EmboTrap was estimated to have per-patient incremental total cost savings of USD $5163 (95% Crl: 4580; 5747) in the US, CAD $2354 (95% Crl: 2153; 2557) in Canada, £1680 (95% Crl: 1571; 1790) in the UK, SEK 31,466 (95% Crl: 29,623; 33,039) in Sweden, €2159 (95% Crl: 2028; 2290) in Germany, €834 (95% Crl: 745; 923) in France, €934 (95% Crl: 855; 1013) in Italy, €3235 (95% Crl: 3068; 3402) in Spain, €2215 (95% Crl: 2073; 2357) in Belgium and €2864 (95% Crl: 2658; 3070) in The Netherlands.

Compared with Solitaire, EmboTrap was estimated to have per-patient incremental total cost savings of USD $6494 (95% Crl: 5900; 7088) in the US, CAD $2962 (95% Crl: 2756; 3169) in Canada, £2114 (95% Crl: 2002; 2226) in the UK, 39,584kr (95% Crl: 37,685; 41,484) in Sweden, €2716 (95% Crl: 2581; 2851) in Germany, €1049 (95% Crl: 959; 1139) in France, €1174 (95% Crl: 1094; 1255) in Italy, €4063 (95% Crl: 3893; 4234) in Spain, €2786 (95% Crl: 2640; 2932) in Belgium and €3583 (95% Crl: 3373; 3793) in The Netherlands (Table 3).

Relative to Solitaire, Trevo was estimated to have per-patient incremental total cost savings of USD $1331 (95% Crl: 722; 1939) in the US, CAD $608 (95% Crl: 395; 820) in Canada, £434 (95% Crl: 317; 550) in the UK, 8118kr (95% Crl: 6139; 10,097) in Sweden, €557 (95% Crl 417; 697) in Germany, €215 (95% Crl 123; 307) in France, €241 (95% Crl: 158; 324) in Italy, €828 (95% Crl: 648; 1009) in Spain, €571 (95% Crl: 420; 723) in Belgium and €719 (95% Crl: 502; 936) in The Netherlands (Table 3).

Across all ten countries, over 99% of iterations found the use of EmboTrap relative to Trevo or Solitaire resulted in per-patient cost savings. The use of Trevo relative to Solitaire was cost saving in over 73% of iterations in all countries (Table 3).

### One-way sensitivity analyses

Across the three pairwise comparisons (EmboTrap versus Solitaire, EmboTrap versus Trevo, Solitaire versus Trevo) in the ten countries assessed, the one-way sensitivity analyses indicated that the results were the most sensitive to variations in the proportion of patients who achieved mRS 0–2 versus mRS 3–5 at 90 days (Supplementary Figures 1–10). Across all countries, the model was less sensitive to variations in the short-term hospitalization LOS, cost of stroke hospital day and 1-year costs for mRS 0–2 and 3–5.

### Scenario analyses

Similar to the probabilistic sensitivity analyses results, the probabilistic scenario analyses estimated that the use of EmboTrap led to the lowest costs, followed by Trevo, then Solitaire. Across all ten countries, the highest per-patient cost savings were observed with the use of EmboTrap relative to Solitaire, followed by the use of EmboTrap relative to Trevo. The use of Trevo relative to Solitaire was associated with the lowest per-patient cost savings. The reported cost savings were reduced relative to the cost savings observed in the deterministic base-case and the probabilistic sensitivity analyses. Furthermore, fewer iterations were associated with costs savings (Supplementary Table 1).

## Discussion

This study assessed the economic impact of improved functional outcomes associated with SR device selection during MT based on MASTRO I [20]. The costs were analyzed from the healthcare system perspective for the US, Canada, the UK, Sweden, Germany, France, Italy, Spain, Belgium and The Netherlands, representing the major Western countries where the three devices are marketed. The results suggest that improved functional outcomes achieved with EmboTrap may translate to lower per-patient total costs and, therefore, cost savings compared with the use of Trevo or Solitaire across all countries studied. The US, followed by Spain, had the highest absolute cost savings for each SR pairwise comparison, whereas France had the lowest. However, when assessing the normalized values of total cost, as demonstrated through percent difference in per-patient total cost, the greatest magnitude of cost savings was observed for Spain, followed by Germany, with France having the lowest magnitude. These results differ from those obtained when comparing the direct absolute values of cost savings, underscoring the nuanced dynamics of cost differentials across health systems and the potential caveats in comparing absolute costs across the countries.

The results of this analysis highlight the potential impact of SR device selection on the economic burden of stroke. Our study also shows that using a SR device with better functional outcomes produced greater cost savings in the long-term versus the short-term in five of the ten countries, highlighting the impact of decision making in an acute care setting, not only on patient outcomes, but also on healthcare resource utilization and costs in the long-term. Our results demonstrate that the economic burden of stroke is not simply a one-time expense associated with acute hospitalization; rather, the economic burden commences with index hospitalization and continues to accumulate over time. Our results also align with Johnson *et al.*, Patel *et al.* and Lucas-Noll *et al.* which highlight the impact of acute care on long-term versus short-term care costs for stroke patients [36–38].

The results of this study also highlight the importance of considering differences between healthcare systems, treatment patterns and costs across various countries. The high total costs estimated for the US are not surprising given that medical costs in the US are among the highest in the world [39]. Resulting total costs in the US were driven by short-term costs, which were roughly 2.5x higher than estimated long-term costs across all three SRs. Germany, Italy, Belgium and The Netherlands also yielded short-term cost results that were higher than long-term costs, but to a lesser magnitude than the US (Table 2). Altogether, these country-specific differences in short- and long-term costs demonstrate that although EmboTrap was associated with cost savings in all countries of interest, the factors driving cost savings vary by country.

MASTRO I found that EmboTrap was associated with improved functional outcomes and reduced mortality at 90 days [20]. Further, per DeWilde *et al.* [24], hospital LOS can correlate with functional outcomes, with patients who achieve 90-day mRS 0–2 having a shorter LOS (8.9 days) compared with patients who achieve mRS 3–5 (18.4 days). This difference in LOS may translate to device-specific differences in healthcare resource use.

The results of this analysis provide valuable insights on SR selection for providers and healthcare policy decision-makers aiming to alleviate the clinical and economic burden of stroke. Though these results should be considered in conjunction with SR prices at the local payer level within each country. Given the healthcare market, disease burden and SR prices, exploring the country-specific cost-effectiveness for SR selection should also be considered. Cost-effectiveness analysis will better assess the long-term value for money given the impact of SR intervention on patients' health and each market's willingness to pay.

Our study has some limitations. First, although best-available costing data were leveraged per country, some assumptions were required to derive costing inputs used in the model. Device prices were not included due to lack of reliable and citable sources for country-specific and product-specific device price, variability of device prices over time and potential variability of prices within each country across different regions. Short-term and long-term cost data were sourced based on country specific calculations, different references, and represent unique healthcare structures. The analysis did not account for cost differences and performance influences associated with the use of rescue devices, aspiration catheters in combination techniques or BGCs. Additionally, the model did not specifically include other stroke-related health issues, such as recurrent stroke, as independent model health states. This is due to the model inputs being based on MASTRO I for mRS score and country-specific studies for cost estimates, in which many did not report this level of detail. Variables such as clot composition, operator skill, hospital volume and accessibility to stroke centers could not be adjusted for; these variables may influence both the clinical and economic outcomes associated with SR use. To derive long-term direct healthcare costs for Germany, France, Canada and The Netherlands, additional manipulations were required (Supplementary Methods). Furthermore, with publication years ranging from 2012 (Italy and Canada) to 2024 (The Netherlands), select sources used to estimate costs may be outdated, may not reflect current clinical practice and/or resource use due to the rapidly evolving stroke treatment landscape, and may not have increased in line with inflation over time. This limitation is underscored by the use of Belgian-specific LOS data from the Dewilde *et al.* study [24]. Together, this highlights the need for ongoing assessment of stroke-related cost data globally, not only to accurately reflect the current stroke-specific burden to healthcare systems but also to improve the precision of economic modeling. As updated cost data for stroke becomes available, the relationship between SR-related functional outcomes and the subsequent economic impact should be reassessed.

The second limitation is that the treatment effect in this model is determined based on reported 90-day mRS score associated with SR selection [20]. The mRS scores were grouped into ‘good’ and ‘poor’ functional outcomes (i.e., mRS 0–2 and 3–5, respectively) and may lack sensitivity in differences at the categorical level (mRS 0–5). Patients with mRS 6 were excluded because quality of life was not considered in this model and cost estimates were not reported for cases resulting in 90-day mortality. Further, mRS is a subjective disability scale commonly used to assess patient outcomes following stroke [13,40] which has inherent strengths and limitations when used to measure the well-being of individuals who have suffered a stroke. While some studies show variability in 90-day mRS, others have demonstrated the validity of mRS [41,42,43]. Studies show 43–76% of patients have unchanged 90-day mRS scores at 12 months [44,45,46,47], indicating that the 90-day mRS score may be a reliable measure of short- and long-term disability. In relation to functional independence, mRS score stability has been demonstrated with the proportion of patients achieving mRS 0–2 at 90-days remaining unchanged for ∼96% of these patients at 12 months [45], thereby supporting the use of 90-day mRS scores in predicting long-term clinical and economic outcomes.

Finally, the difference in economic outcomes demonstrated between the commonly used SRs evaluated (EmboTrap, Trevo and Solitaire) in the current model may be influenced by the geometric configuration of the SR [19]. Studies have demonstrated differences in recanalization outcomes between SRs, which may impact patient functional outcomes following MT [20,21,22]. EmboTrap is a third-generation dual-layer nitinol SR with a closed cell inner channel with a high radial force that is surrounded by an open-cell configuration for instant recanalization and a closed-cell configuration on the distal tip to catch clot fragments [19]. Trevo and Solitaire are both second-generation nitinol stents with a closed cell, barrel, peak-to-peak geometric configuration designed to leverage radial force alone to capture and remove the clot [19]. The difference in device design between EmboTrap, Trevo and Solitaire may influence the observed improvements in functional and, subsequently, economic outcomes. Data was not available to evaluate differences in clinical and economic outcomes for other third-generation SRs, such as Tigertriever (Rapid Medical), 3D Revascularization Device (Penumbra) and Eric (Microvention) [19].

Overall, this study sought to assess the relationship between SR-related functional outcomes and their subsequent economic impact following MT, and assumed all other procedural techniques were similar across cohorts. The results of MASTRO I suggest that SR choice may impact post-stroke patient functional outcomes, as demonstrated by EmboTrap's association with higher rates of functional independence followed by Trevo and then Solitaire [20]. The current study demonstrates that improved functional outcomes may be associated with lower healthcare costs and therefore cost savings, thereby reducing the clinical and economic burden of stroke across countries in Europe and North America. Overall, the results of the analysis support the importance of SR selection during MT to optimize clinical and economic outcomes in the short and long-term across healthcare systems.

## Conclusion

Across the ten countries studied in this model, patients with AIS treated with EmboTrap alone on the first attempt were estimated to have the lowest healthcare costs, followed by those treated with Trevo, with those treated with Solitaire having the highest costs among patients who survived to 90 days. Based on total cost, the greatest cost savings over the 1-year study period were reported for EmboTrap versus Solitaire, followed by EmboTrap versus Trevo, with the smallest cost savings reported between Trevo versus Solitaire. The cost savings reported in this analysis are attributable to the lower healthcare resource utilization for patients treated with EmboTrap who had the highest rates of good functional outcomes (mRS 0–2). With rising healthcare costs and limited hospital budgets, these results suggest that EmboTrap proves to be an evidence-based economical choice of stent retriever for hospitals and healthcare systems.

## Summary points

- The economic impact of achieving improved functional outcomes was explored across three commonly used stent retrievers (SRs; EmboTrap, Trevo and Solitaire) using functional outcome data reported in MASTRO I, a recent living systematic review and meta-analysis.
- Based on the proportion of patients who achieved a 90-day modified Rankin Scale score of 0–2 as reported in MASTRO I, costs associated with short-term index hospitalization, long-term one-year post-hospitalization costs and total costs (short-term index hospitalization and 1-year post-hospitalization costs) were estimated for each device.
- Total (short- and long-term) cost savings were reported from the perspective of the US, Canada, the UK, Sweden, Germany, France, Italy, Spain, Belgium and The Netherlands healthcare systems.
- Across all countries studied, patients treated with EmboTrap during mechanical thrombectomy (MT) were associated with the lowest costs, followed by patients treated with Trevo, and then those treated with Solitaire. The reduced costs were likely due to the shorter length of stay and the lower healthcare resource use 1-year post-stroke associated with achieving 90-day good functional outcomes.
- Over the 1-year period, the greater savings in total cost were reported for EmboTrap versus Solitaire, followed by EmboTrap versus Trevo, with Trevo versus Solitaire having the smallest cost savings.
- In summary, the use of EmboTrap resulted in the lowest per-patient costs, resulting in cost savings relative to Trevo and Solitaire, due to the proportion of patients achieving good functional outcomes.
- The results of this analysis provides additional information that payers and clinicians may leverage in making evidence-based decisions to inform SR selection during MT.
- The results of this study should be interpreted in the context of the limitations of the analysis including heterogeneity in the MASTRO I dataset, the inability to account for all potential variables that may influence MT costs, use of potentially outdated economic data, and that adjunctive device use was not able to be fully evaluated in the analysis.
- Further research is needed that examines the relationship between functional outcomes and short- and long-term costs in stroke treatment.

## Supplementary Material


