# Supplementary material for: The economic impact of stent retriever selection for acute ischemic stroke: a cost analysis of MASTRO I from the healthcare system perspective of the United States, Canada and eight European countries
Source: J Comp Eff Res. 2025 Feb 17;14(3):e240216. doi: 10.57264/cer-2024-0216 (PMC11864083; doi:10.57264/cer-2024-0216)
Supplement: Supplementary file 1 [file cer-14-240216-s1.docx]

# Supplementary Tables and Figures

Supplementary Table 1. Economic Outcomes of Probabilistic Scenario Analyses

| **Device** | **Short-Term Cost**  **Mean [95% CrI]** | **Long-Term Cost**  **Mean [95% CrI]** | **Total Cost**  **Mean [95% CrI]** | **Per-Patient Incremental Cost Compared to EmboTrap**  **Mean [95% CrI]** | **Iterations with Cost Savings Using EmboTrap** | **Per-Patient Incremental Cost Compared to Trevo**  **Mean [95% CrI]** | **Iterations with Cost Savings Using Trevo** |
| --- | --- | --- | --- | --- | --- | --- | --- |
| **US (USD)** | | | | | | | |
| EmboTrap | $66,734  [66,608; 66,860] | $26,290  [26,259; 26,322] | $93,024  [92,893; 93,155] | - | - |  |  |
| Trevo | $68,382  [68,252; 68,512] | $27,361  [27,327; 27,394] | $95,743  [95,608; 95,879] | $2,719  [2,123; 3,315] | 91.54% | - | - |
| Solitaire | $69,689  [69,557; 69,822] | $28,212  [28,177; 28,247] | $97,901  [97,763; 98,039] | $4,878  [4,276; 5,479] | 99.37% | $2,158  [1,547; 2,770] | 85.47% |
| **Canada (CAD)** | | | | | | | |
| EmboTrap | $16,152  [16,122; 16,183] | $29,074  [29041; 29107] | $45,226  [45181; 45271] | - | - |  |  |
| Trevo | $16,551  [16,520; 16,583] | $29,914  [29880; 29948] | $46,465  [46418; 46512] | $1,239 [1032; 1446] | 91.54% | - | - |
| Solitaire | $16,868  [16,836; 16,900] | $30,582  [30547; 30618] | $47,450  [47401; 47498] | $2,224 [2014; 2433] | 99.37% | $985 [771; 1198] | 85.47% |
| **UK (GBP)** | | | | | | | |
| EmboTrap | £9,409  [9,391; 9,427] | £11,937  [11,921; 11,953] | £21,346  [21,321; 21370] | - | - | - | - |
| Trevo | £9,641  [9,623; 9,660] | £12,589  [12,572; 12,606] | £22,230  [22,204; 22,256] | £884  [772; 997] | 91.54% | - | - |
| Solitaire | £9,826  [9,807; 9,844] | £13,107  [13,089; 13,125] | £22,933  [22,906; 22,959] | £1,587  [1,473; 1,701] | 99.37% | £703  [586; 820] | 85.47% |
| **Sweden (SEK)** | | | | | | | |
| EmboTrap | 142,213kr  [141,942; 142,485] | 212,155kr  [211,850; 212,449] | 354,357kr  [353,950; 354,775] | - | - |  |  |
| Trevo | 145,729kr  [145,447; 146,001] | 225,192kr  [224,875;225,519] | 370,921kr  [370,921; 371,362] | 16,654kr  [14,653;18,464] | 91.54% | - | - |
| Solitaire | 148,511kr  [148,228;148,794] | 235,571kr  [235,232;235,910] | 384,082kr  [383,630; 384,534] | 29,725kr  [27,780;31,658] | 99.37% | 13,161kr  [11,171;15,151] | 85.47% |
| **Germany (EUR)** | | | | | | | |
| EmboTrap | €11,669  [11,647; 11,691] | €11,807  [11,789; 11,826] | €23,476  [23,447; 23,505] | - | - |  |  |
| Trevo | €11,957  [11,934; 11,980] | €12,655  [12,636; 12,675] | €24,612  [24,581; 24,643] | €1,136  [1,001; 1,271] | 91.54% | - | - |
| Solitaire | €12,186  [12,163; 12,209] | €13,330  [13,309; 13,351] | €25,515  [25,484; 25,547] | €2,039  [1,902; 2,177] | 99.37% | €903  [762; 1044] | 85.47% |
| **France (EUR)** | | | | | | | |
| EmboTrap | €8,475  [8,459; 8,491] | €10,501  [10,489; 10,512] | €18,976  [18,956; 18,996] | - | - |  |  |
| Trevo | €8,685  [8,668; 8,701] | €10,730  [10,719; 10,742] | €19,415  [19,395; 19,436] | €439  [349; 529] | 91.54% | - | - |
| Solitaire | €8,851  [8,834; 8,867] | €10,913  [10,901; 10,925] | €19,764  [19,743; 19,785] | €788  [696; 879] | 99.37% | €349  [256; 441] | 85.47% |
| **Italy (EUR)** | | | | | | | |
| EmboTrap | €8,438  [8,422; 8,454] | €5,399  [5,392; 5,406] | €13,838  [13,820; 13,855] | - | - |  |  |
| Trevo | €8,647  [8,630; 8,663] | €5,682  [5,675; 5,690] | €14,329  [14,311; 14,348] | €492  [411; 572] | 91.54% | - | - |
| Solitaire | €8,812  [8,795; 8,829] | €5,907  [5,899; 5,915] | €14,720  [14,701; 14,738] | €882  [800; 964] | 99.37% | €390  [307; 474] | 85.47% |
| **Spain (EUR)** | | | | | | | |
| EmboTrap | €9,318  [9,301; 9,336] | €20,175  [20,144; 20,206] | €29,494  [29,457; 29,530] | - | - |  |  |
| Trevo | €9,544  [9,526; 9,562] | €21,633  [21,599; 21,666] | €31,177  [31,138; 31,216] | €1,683  [1,514; 1,852] | 91.59% | - | - |
| Solitaire | €9,730  [9,712; 9,748] | €22,823  [22,788; 22,858] | €32,553  [32,512; 32,594] | €3,059  [2,887; 3,232] | 99.12% | €1,376  [1,198; 1,555] | 85.75% |
| **Belgium (EUR)** | | | | | | | |
| EmboTrap | €12,627  [12,603; 12,651] | €14,320  [14,300;14,340] | €26,947  [26,915;26,979] | - | - |  |  |
| Trevo | €12,939  [12,914; 12,963] | €15,174  [15,152;15,195] | €28,112  [28,079;28,146] | €1,166  [1,019; 1,312] | 91.54% | - | - |
| Solitaire | €13,186  [13,161; 13,211] | €15,853  [15,830;15,875] | €29,039  [29,004;29,073] | €2,092  [1,943; 2,241] | 99.37% | €926  [774; 1,078] | 85.47% |
| **The Netherlands (EUR)** | | | | | | | |
| EmboTrap | €20,608  [20,570; 20,647] | €18,524  [18,500; 18,549] | €39,133  [39,085; 39,180] | - | - |  |  |
| Trevo | €21,108  [21,069; 21,148] | €19,505  [19,478; 19,531] | €40,613  [40,564; 40,662] | €1,480  [1,265; 1,696] | 91.25% | - | - |
| Solitaire | €21,517  [21,477; 21,558] | €20,309  [20,281; 20,336] | €41,826  [41,775; 41,877] | €2,694  [2,474; 2,913] | 99.22% | €1,213  [990; 1,437] | 86.05% |

Probabilistic analyses were based on parametric Monte Carlo simulations with 10,000 iterations.

Note: Results for Germany represent 2023 and 2024 values due to the use of 2024 physician fees to calculate long-term costs.

Abbreviations: CrI = credible interval; CAD = Canadian dollar; GBP = Great Britain Pound; EUR = Euro; mRS = modified Rankin Scale at 90 days post-operative; SEK = Swedish krona; UK = United Kingdom; US = United States; USD = United States dollar.

Supplementary Figure 1. Tornado Diagrams for Per-Patient Incremental Cost - US Analysis


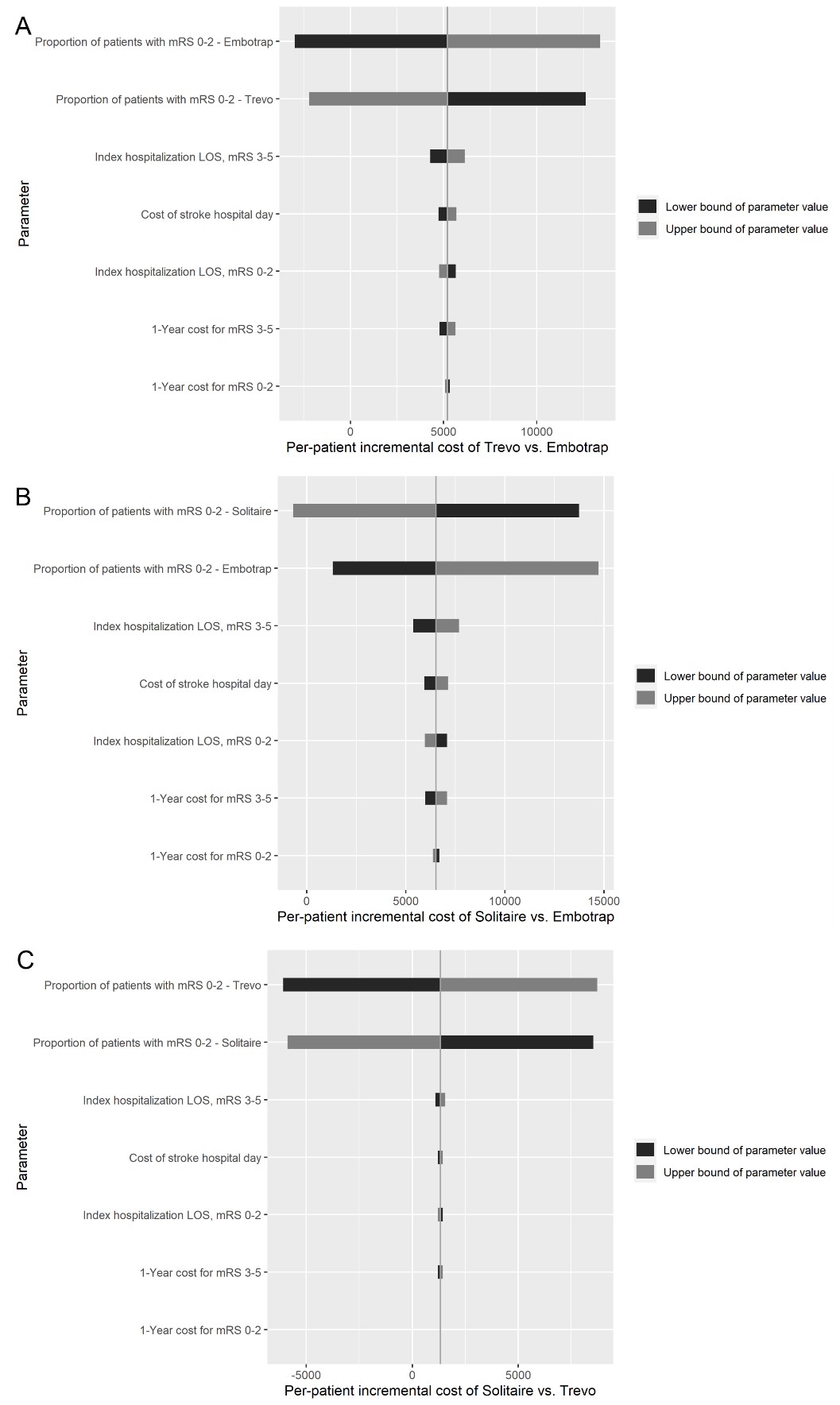


Tornado diagram comparing for per-patient incremental cost of (A) Trevo versus EmboTrap, (B) Solitaire versus EmboTrap, and (C) Solitaire versus Trevo.

Abbreviations: LOS = length of stay; mRS = modified Rankin Scale at 90 days post-operative; US = United States.

Supplementary Figure 2. Tornado Diagrams for Per-Patient Incremental Cost - Canada Analysis


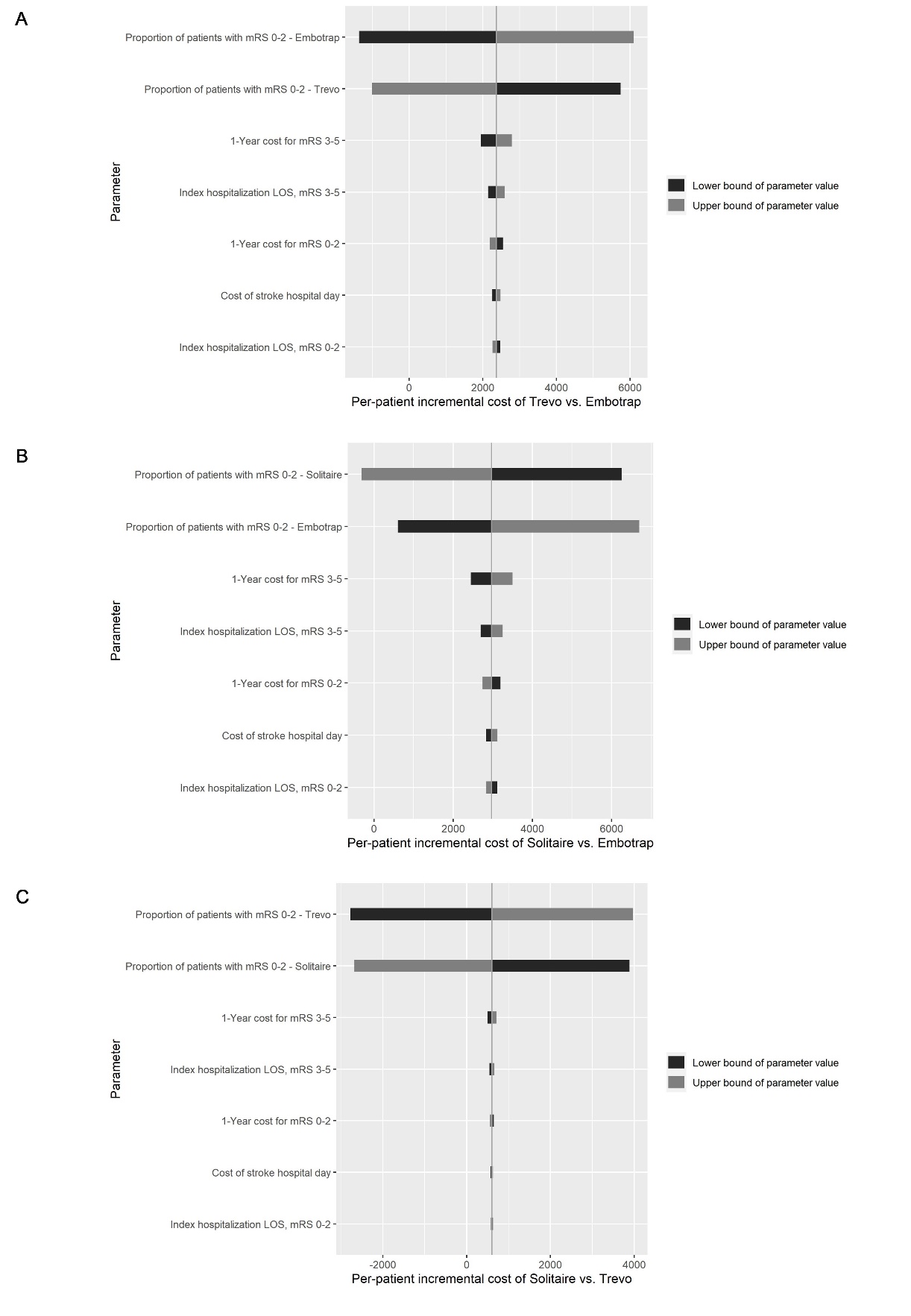


Tornado diagram comparing for per-patient incremental cost of (A) Trevo versus EmboTrap, (B) Solitaire versus EmboTrap, and (C) Solitaire versus Trevo.

Abbreviations: LOS = length of stay; mRS = modified Rankin Scale at 90 days post-operative.

Supplementary Figure 3. Tornado Diagrams for Per-Patient Incremental Cost - UK Analysis


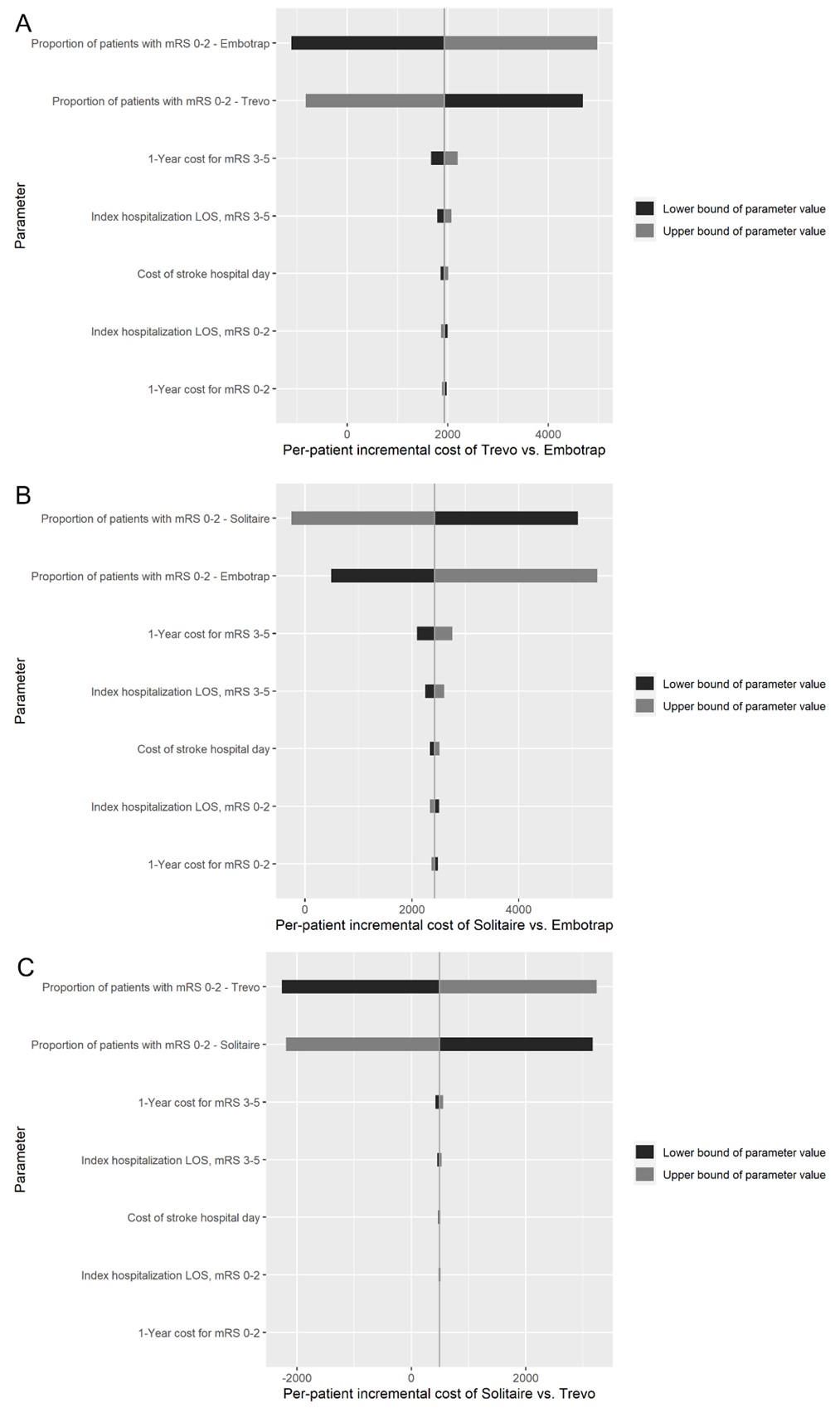


Tornado diagram comparing for per-patient incremental cost of (A) Trevo versus EmboTrap, (B) Solitaire versus EmboTrap, and (C) Solitaire versus Trevo.

Abbreviations: LOS = length of stay; mRS = modified Rankin Scale at 90 days post-operative; UK = United Kingdom.

Supplementary Figure 4. Tornado Diagrams for Per-Patient Incremental Cost - Sweden Analysis


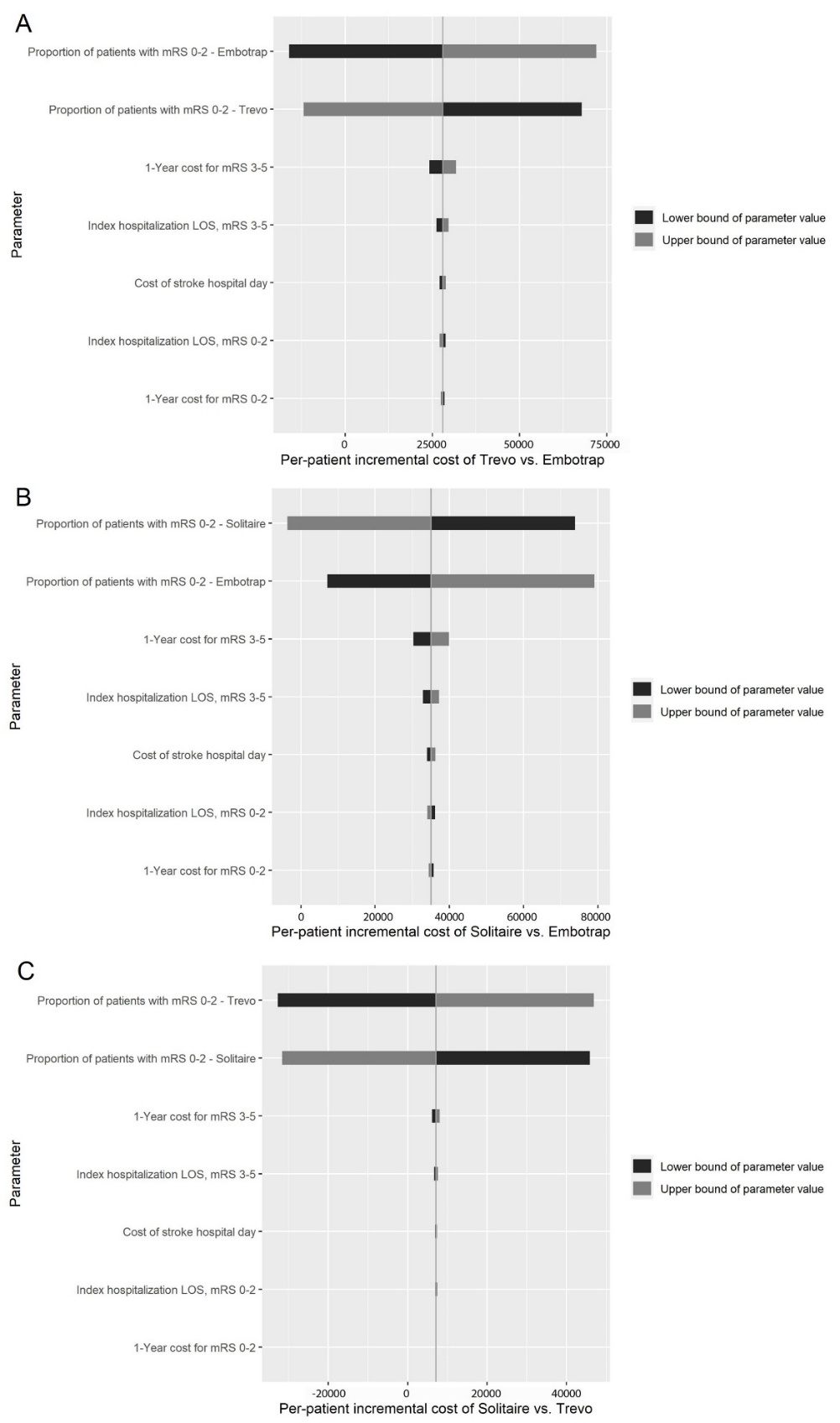


Tornado diagram comparing for per-patient incremental cost of (A) Trevo versus EmboTrap, (B) Solitaire versus EmboTrap, and (C) Solitaire versus Trevo.

Abbreviations: LOS = length of stay; mRS = modified Rankin Scale at 90 days post-operative.

Supplementary Figure 5. Tornado Diagrams for Per-Patient Incremental Cost - Germany Analysis


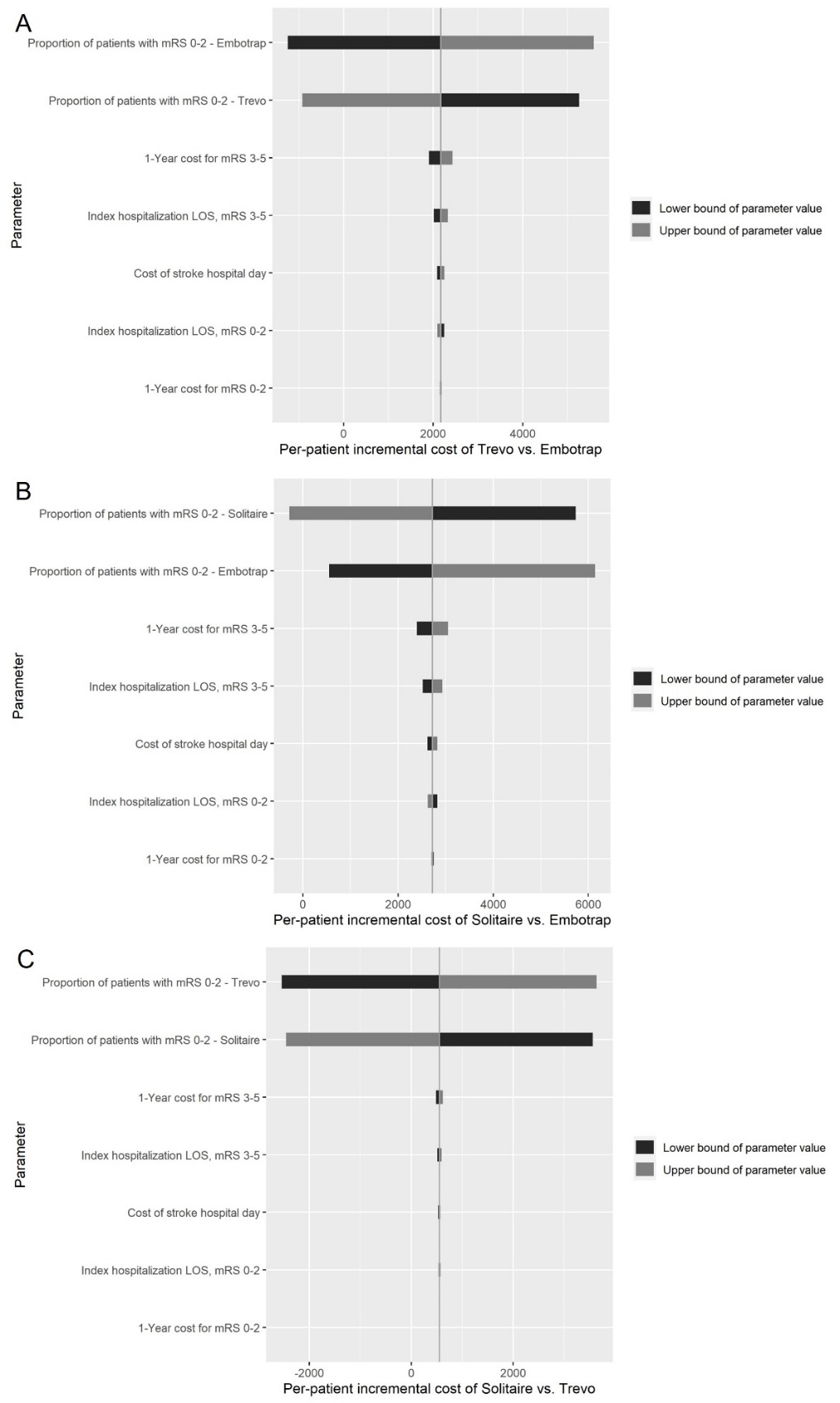


Tornado diagram comparing for per-patient incremental cost of (A) Trevo versus EmboTrap, (B) Solitaire versus EmboTrap, and (C) Solitaire versus Trevo.

Abbreviations: LOS = length of stay; mRS = modified Rankin Scale at 90 days post-operative.

Supplementary Figure 6. Tornado Diagrams for Per-Patient Incremental Cost - France Analysis


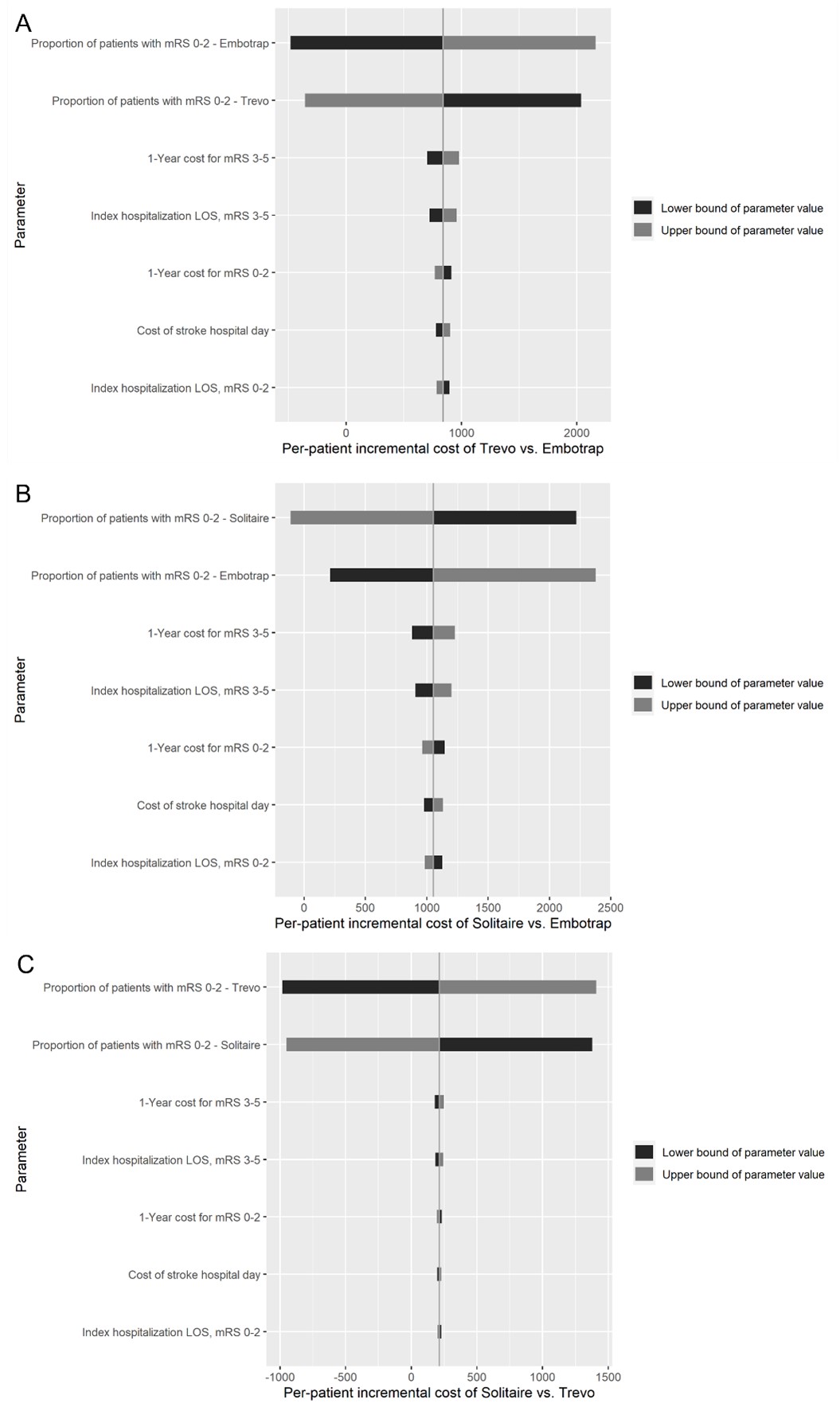


Tornado diagram comparing for per-patient incremental cost of (A) Trevo versus EmboTrap, (B) Solitaire versus EmboTrap, and (C) Solitaire versus Trevo.

Abbreviations: LOS = length of stay; mRS = modified Rankin Scale at 90 days post-operative.

Supplementary Figure 7. Tornado Diagrams for Per-Patient Incremental Cost - Italy Analysis


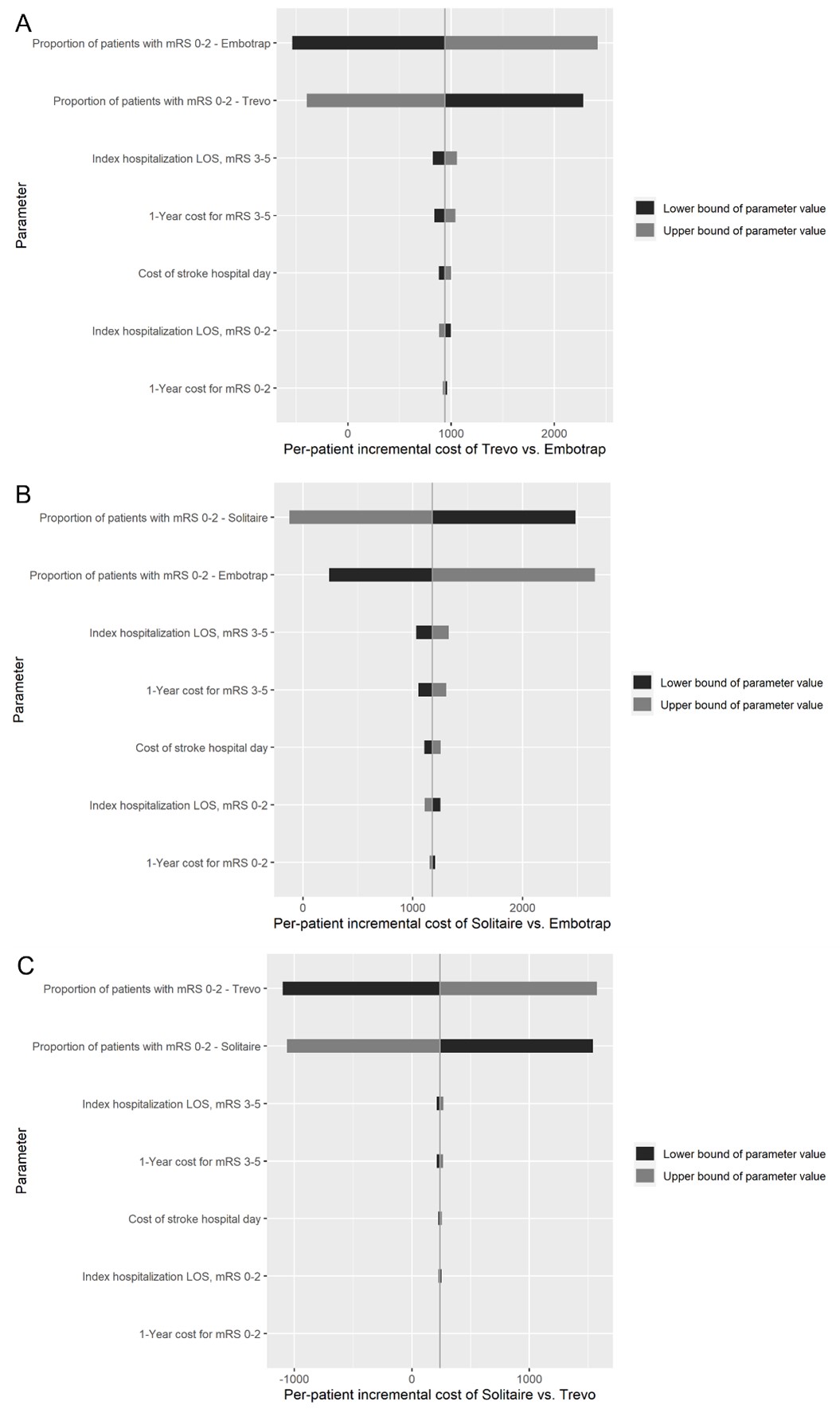


Tornado diagram comparing for per-patient incremental cost of (A) Trevo versus EmboTrap, (B) Solitaire versus EmboTrap, and (C) Solitaire versus Trevo.

Abbreviations: LOS = length of stay; mRS = modified Rankin Scale at 90 days post-operative.

Supplementary Figure 8. Tornado Diagrams for Per-Patient Incremental Cost - Spain Analysis


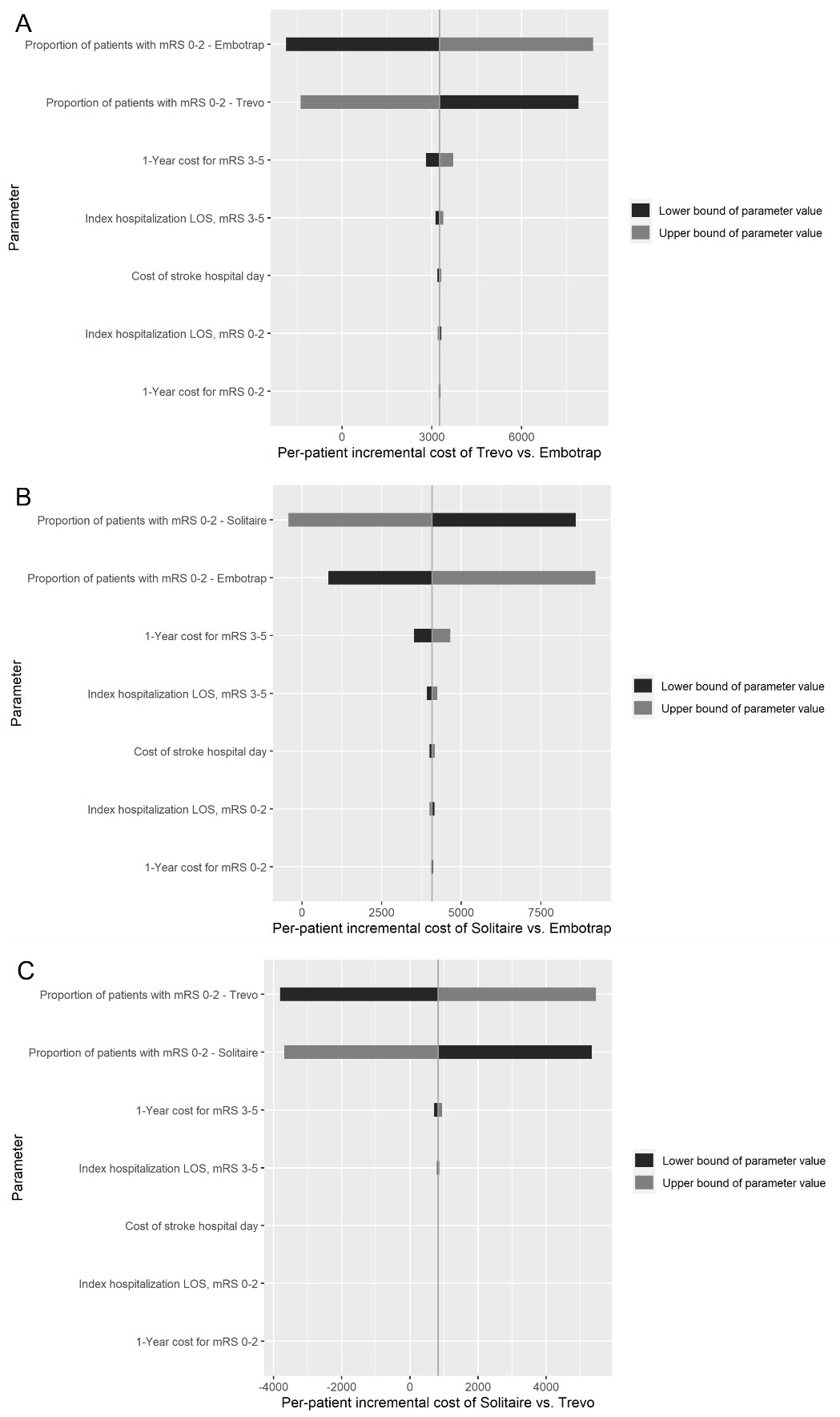


Tornado diagram comparing for per-patient incremental cost of (A) Trevo versus EmboTrap, (B) Solitaire versus EmboTrap, and (C) Solitaire versus Trevo.

Abbreviations: LOS = length of stay; mRS = modified Rankin Scale at 90 days post-operative.

Supplementary Figure 9. Tornado Diagrams for Per-Patient Incremental Cost - Belgium Analysis


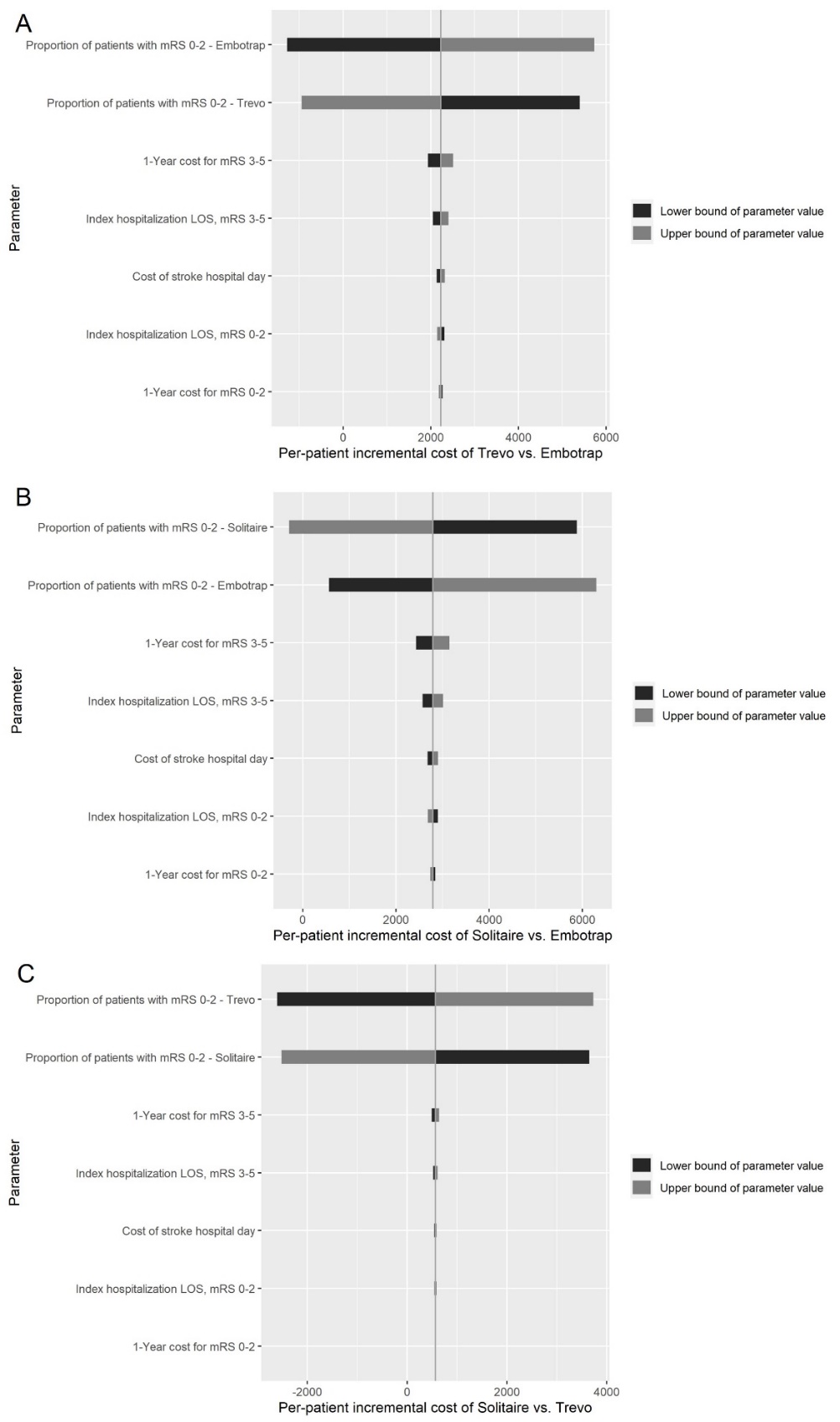


Tornado diagram comparing for per-patient incremental cost of (A) Trevo versus EmboTrap, (B) Solitaire versus EmboTrap, and (C) Solitaire versus Trevo.

Abbreviations: LOS = length of stay; mRS = modified Rankin Scale at 90 days post-operative.

Supplementary Figure 10. Tornado Diagrams for Per-Patient Incremental Cost – The Netherlands Analysis


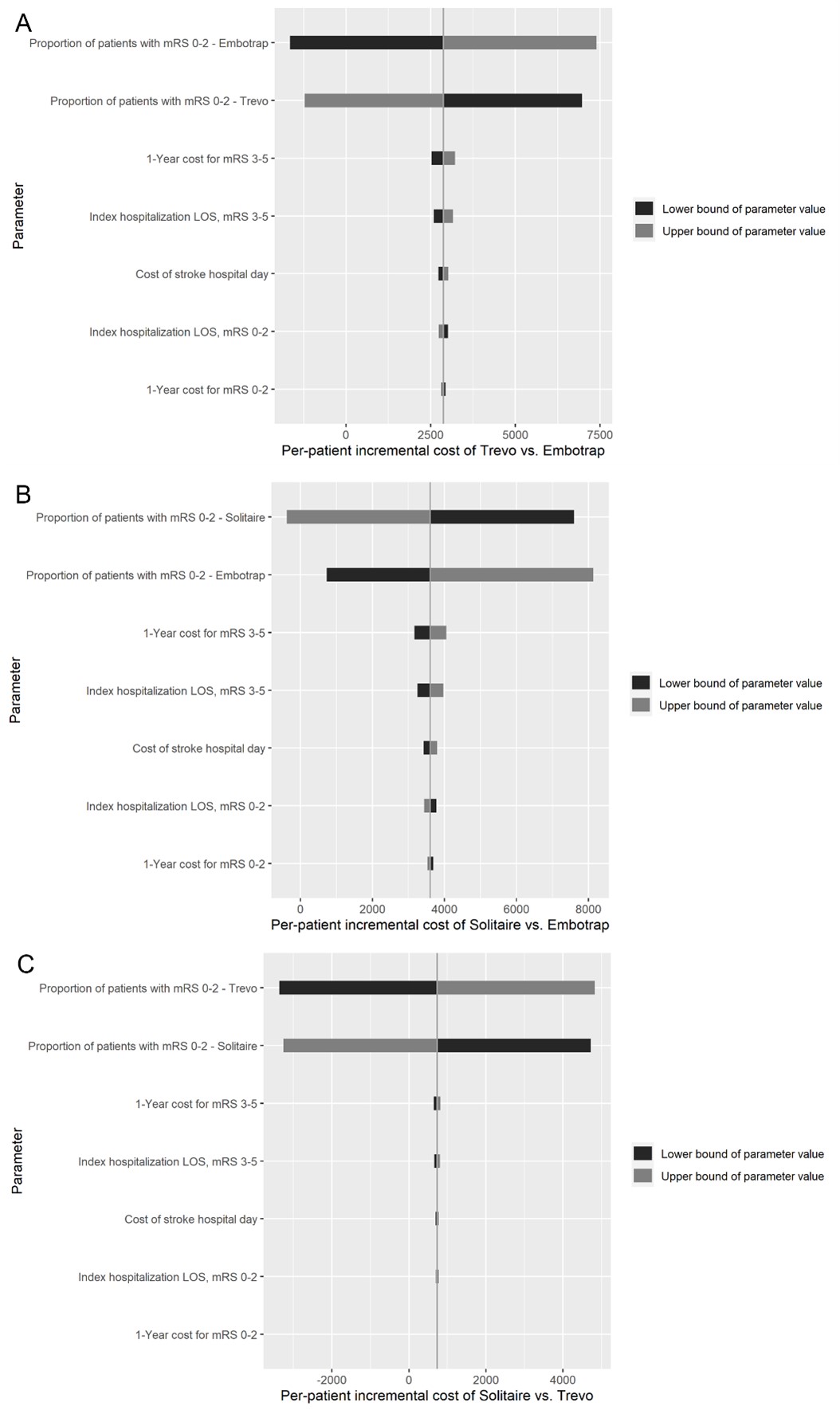


Tornado diagram comparing for per-patient incremental cost of (A) Trevo versus EmboTrap, (B) Solitaire versus EmboTrap, and (C) Solitaire versus Trevo.

Abbreviations: LOS = length of stay; mRS = modified Rankin Scale at 90 days post-operative

# Supplementary Methods

**Cost Inputs for Canada**

Cost per stroke hospital day, short-term and total costs were available for Canada in Mittman et al. 2012 [1]; however, long-term costs defined in this model (i.e. one-year post stroke costs) were not reported. Hence, the input value for long-term costs for mRS 0-2 and 3-5 were calculated as total costs (Mittman et al. 2015) less LOS-based short-term calculated costs, as defined for this study.

**Cost Inputs for Germany**

A recent publication by Oliveira Gonçalves et al. 2023 [2] reported long-term nursing care costs associated with mRS 0-5 levels in Germany based on three residential care settings: living at home without professional care, living at home with professional care, and not living at home (i.e., full-time institutional residence). Employing the methodology outlined by Oliveira Gonçalves et al. 2023, the 12-month post-stroke cost per mRS 0-5 was extracted and updated using 2024 rates for national care fees [3-5]. Using data from the Official National Statistics Distribution of the Population in Need of Care by Residential Status [6], the national distribution and proportion of Germans in need of care living in each of the residential statuses in 2021 was calculated and used to impute a weighted average 12-month post-stroke cost per mRS level. Given this cost data only comprised long-term social care costs, which is a limitation, OECD data was used to source the proportion of the gross domestic product (GDP) that Germany spends on healthcare and the proportion of the GDP spent on long-term care to estimate the total annual healthcare cost (direct healthcare and social care costs) for Germany in 2024 Euros [7].

**Cost Inputs for France**

Barral et al. 2020 [8] is a cost-effectiveness analysis comparing SR thrombectomy combined with standard of care (intravenous thrombolysis) versus standard of care alone from a societal perspective in France. The study reported post-stroke direct medical costs (initial hospital stay, procedure, SR, recurrent hospital stay, rehabilitation hospital stay, medical and paramedical visits, medication, and biological tests), direct non-medical costs (professional care, informal care, nursing home), and indirect costs (patient productivity losses with a professional activity) by mRS categorized in an acute phase (0-7 days post-stroke), 8-90 days post-stroke, and in the 90-days to 1 year post-stroke. To ensure consistency with the healthcare system perspective indirect costs, for example patient productivity losses, were not included in the analysis. Some input costs are based on government regulated DRG values and these may not increase in line with inflation, which may limit interpretability of findings.

**Cost Inputs for The Netherlands**

Pinckaers et al. 2024 [9] reported estimates for utilities and costs up to two years post-stroke based on 90-day mRS score from a Dutch societal perspective. We consulted with the primary author (F.M.E. Pinckaers) to clarify the study reported cost estimations described as “12-month care cost” in the article. The author confirmed these represented the previous 3-months costs within the 12-months post-stroke timeframe. Therefore, source costs were multiplied by four to estimate a total 1-year post-stroke cost, according to mRS level, for The Netherlands.

# Supplementary Material References

1. Mittmann N, Seung SJ, Hill MD *et al.* Impact of disability status on ischemic stroke costs in Canada in the first year. *Can J Neurol Sci* 39(6), 793-800 (2012).

2. Oliveira Gonçalves ASO, Rohmann JL, Piccininni M *et al.* Economic Evaluation of a Mobile Stroke Unit Service in Germany. *Ann Neurol* 93(5), 942-951 (2023).

3. Bundesministerium für Gesundheit. Pflegegeld. 2024.

4. Bundesministerium für Gesundheit. Ambulante Pflegesachleistungen. 2024.

5. Bundesministerium für Gesundheit. Vollstationäre Pflege im Heim. 2024.

6. Destatis Statistisches Bundesamt. Pflegestatistik - Pflege im Rahmen der Pflegeversicherung - Deutschlandergebnisse - 2021. 2022.

7. OECD. Health at a Glance 2023.

8. Barral M, Armoiry X, Boudour S *et al.* Cost-effectiveness of stent-retriever thrombectomy in large vessel occlusion strokes of the anterior circulation: Analysis from the French societal perspective. *Rev Neurol (Paris)* 176(3), 180-188 (2020).

9. Pinckaers FME, Grutters JPC, Huijberts I *et al.* Cost and Utility Estimates per Modified Rankin Scale Score up to 2 Years Post Stroke: Data to Inform Economic Evaluations From a Societal Perspective. *Value Health* 27(4), 441-448 (2024).
